# Structure of Epstein-Barr virus tegument protein complex BBRF2-BSRF1 reveals its potential role in viral envelopment

**DOI:** 10.1038/s41467-020-19259-x

**Published:** 2020-10-26

**Authors:** Hui-Ping He, Meng Luo, Yu-Lu Cao, Yu-Xin Lin, Hua Zhang, Xiao Zhang, Jun-Ying Ou, Bing Yu, Xiaoxue Chen, Miao Xu, Lin Feng, Mu-Sheng Zeng, Yi-Xin Zeng, Song Gao

**Affiliations:** 1 State Key Laboratory of Oncology in South China, Collaborative Innovation Center for Cancer Medicine, Sun Yat-sen University Cancer Center, 510060 Guangzhou, China; 2 Molecular Imaging Center, Guangdong Provincial Key Laboratory of Biomedical Imaging, the Fifth Affiliated Hospital, Sun Yat-sen University, 519000 Zhuhai, China; 3 Guangzhou Regenerative Medicine and Health Guangdong Laboratory, 510530 Guangzhou, China

**Keywords:** X-ray crystallography, Peptide nucleic acid oligo, Herpes virus

## Abstract

Epstein-Barr virus (EBV) is a γ-herpesvirus associated with the occurrence of several human malignancies. BBRF2 and BSRF1 are two EBV tegument proteins that have been suggested to form a hetero-complex and mediate viral envelopment, but the molecular basis of their interaction and the functional mechanism of this complex remains unknown. Here, we present crystal structures of BBRF2 alone and in complex with BSRF1. BBRF2 has a compact globular architecture featuring a central β-sheet that is surrounded by 10 helices, it represents a novel fold distinct from other known protein structures. The central portion of BSRF1 folds into two tightly associated antiparallel α-helices, forming a composite four-helix bundle with two α-helices from BBRF2 via a massive hydrophobic network. In vitro, a BSRF1-derived peptide binds to BBRF2 and reduces the number of viral genome copies in EBV-positive cells. Exogenous BBRF2 and BSRF1 co-localize at the Golgi apparatus. Furthermore, BBRF2 binds capsid and capsid-associated proteins, whereas BSRF1 associates with glycoproteins. These findings indicate that the BBRF2-BSRF1 complex tethers EBV nucleocapsids to the glycoprotein-enriched Golgi membrane, facilitating secondary envelopment.

## Abstract (web summary)

Epstein-Barr virus (EBV) tegument proteins BBRF2 and BSRF1 have been suggested to form a hetero-complex. Here, He et al. provide the crystal structures of BBRF2 alone and in complex with BSRF1 and suggest that the complex tethers EBV nucleocapsids to the Golgi membrane, facilitating secondary envelopment.

## Introduction

Epstein-Barr virus (EBV) is a double-stranded DNA virus that is carried by ~95% of the human population. EBV infects B lymphocytes and epithelial cells, and can persist throughout life with alternate periods of latency state and lytic replication^1,2^. EBV infection usually results in a mild, if any, pathogenic effect in healthy individuals. In a few cases, it causes acute infectious mononucleosis, particularly upon the first exposure in adolescents and adulthood^3^. In immunosuppressed individuals, such as AIDS patients and transplant recipients, EBV can cause fatal lymphoproliferative disease^4^. Another life-threatening issue of EBV is that it is an oncogenic virus closely associated with several human malignancies, including Burkitt’s lymphoma, Hodgkin’s lymphoma, T/NK cell lymphoma, gastric carcinoma, and nasopharyngeal carcinoma^5,6^. Currently, no effective clinical approach is available to prevent or eliminate EBV infection.

EBV belongs to the Herpesviridae family, which is comprised of α-, β-, and γ-subfamilies^7^. Representatives of the ɑ-herpesvirinae are HSV-1 and HSV-2, which are common human-infecting pathogens that have been extensively studied. The β-herpesvirus subfamily includes human cytomegalovirus (HCMV). EBV and Kaposi’s Sarcoma (KSHV) are members of the γ-herpesvirinae. Herpesviruses share a common virion morphology and feature two steps of envelopment before egress. The nascent herpesvirus nucleocapsids assembled in host cell nuclei translocate to the cytoplasm via primary envelopment/de-envelopment at the nuclear membrane^8^. These capsids then undergo a secondary envelopment to acquire envelope from host organelles, particularly the Golgi apparatus where the viral glycoproteins reside^9^. The exact mechanism of herpesvirus secondary envelopment is not fully understood.

In herpesviruses, the space between the nucleocapsid and the envelope is called the tegument. The EBV genome contains over 80 reading frames, more than 10 of which encode proteins that reside in the tegument and, thus, are termed tegument proteins^5,10^. Tegument proteins are engaged in viral morphogenesis, envelopment, egress, evasion from host immune surveillance, and in preparing and reprogramming the cell for viral replication^11^. Other viral gene products including the surface glycoproteins and transcription factors have been studied extensively^12,13^, but structural information and functional mechanisms for the majority of EBV tegument proteins, including BBRF2 and BSRF1, remain elusive.

BBRF2 is an EBV tegument protein that has putative homologues in all three herpesvirus subfamilies^10^. BBRF2 was recently shown to be a binding partner of BSRF1, another poorly characterized EBV tegument protein^14^. Complexing of the two proteins alters the subcellular localization of BBRF2, and prevents BSRF1 from degradation, which ultimately augments viral infectivity^15^. The BBRF2 homologue in KSHV, ORF42, has been shown to be required for efficient production of viral particles, but is dispensable for reactivation of the lytic cycle^16^. Tegument proteins pUL7 in HSV and pUL103 in HCMV, which are homologues of BBRF2 in α- and β-herpesviruses, have been reported to have similar roles in viral assembly and egress^17,18^. These BBRF2 homologues in three herpesvirus subfamilies also exhibit other specific functions. ORF42 appears to post-transcriptionally regulate the expression of some viral genes in KSHV^16^. The BBRF2-BSRF1 interaction seems to be conserved across herpesviruses, as homologous interactions have been reported for HSV-1 (pUL7-pUL51)^12^ and HCMV (pUL103-pUL71)^19^. In HSV-1, the pUL7-pUL51 complex is required to promote the assembly and cell-to-cell spread of virions, and potentially also to stabilize focal adhesions^12,17,20^. However, the exact function of EBV BBRF2 is not fully understood, and the molecular basis of the interaction between BBRF2 and BSRF1 is unclear, partly due to the lack of structural information.

Here, we report crystal structures of BBRF2 alone and in complex with BSRF1. BBRF2 has a compact globular architecture that represents a novel fold different from other known protein structures. The central part of BSRF1 folds into two tightly associated antiparallel α-helices, which form a heterogeneous four-helix bundle with two α-helices from BBRF2 via hydrophobic interactions. This interface can be disrupted by a BSRF1-derived peptide. The BBRF2-BSRF1 complex resides on the Golgi apparatus. BBRF2 binds EBV capsid protein MCP and capsid-associated protein BPLF1, whereas BSRF1 associates with EBV glycoproteins gB and gH/gL. These data elucidate the molecular basis of the functional association between BBRF2 and BSRF1, suggesting a role of BBRF2-BSRF1 in tethering EBV nucleocapsids to the Golgi membrane during secondary envelopment.

## Results

### Overall structure of BBRF2

**Fig. 1:**
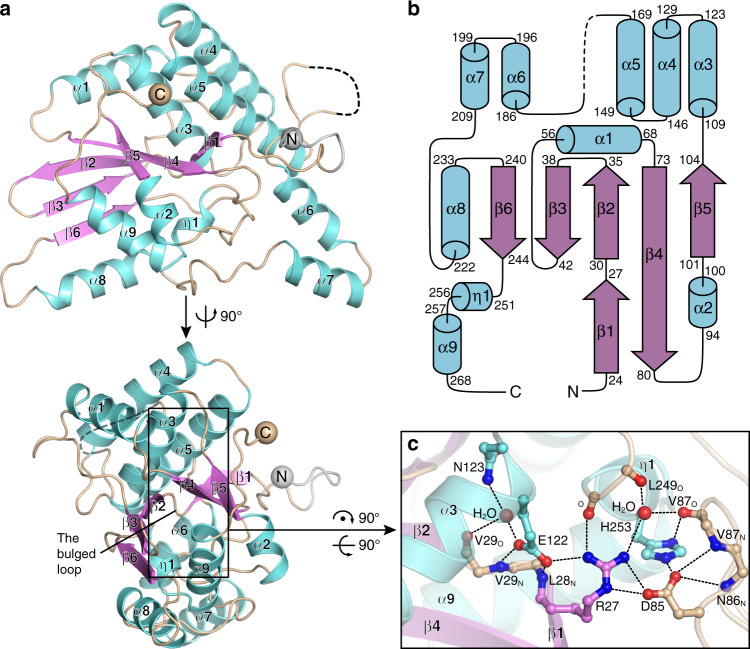
Overall structure of BBRF2Δ. **a** Structure of BBRF2Δ. Helices and β-strands are specified and coloured cyan and magenta, respectively. The vector encoded residues are coloured grey. The N- and C-termini are indicated by spheres. The vector-encoding part of the N-terminal extension is coloured grey. The disordered loop is shown as a dashed line. **b** Topology diagram of BBRF2Δ. Secondary structural elements are not drawn to scale, and are coloured as in a. The boarders of the elements are specified. **c** Details of the hydrophilic core of BBRF2Δ, with α4 and α5 removed for clarity. The main chain oxygen and nitrogen atoms involved in the contact are indicated in subscript with the corresponding amino acids.

To obtain functional insights into BBRF2, we recombinantly expressed the protein in *Escherichia coli* and purified it. BBRF2 is a monomer in solution (Supplementary Fig. 1a). As crystallization of full-length BBRF2 was not successful, we modified the construct and determined the structure of an N- terminally truncated version with deletion of residues 1−16 (BBRF2Δ) at 1.6 Å. The phases were obtained by single anomalous diffraction using selenomethionine (SeMet) derivative, and the final model was refined to an *R*_free_ of 0.189 (Table 1). ΒΒRF2Δ is monomeric both in solution and in the asymmetric unit of the crystal (Supplementary Fig. 1b). Residues of the whole crystallized construct, including those encoded by the vector sequence, except for 172–178, are clearly seen in the ΒΒRF2Δ structure (Fig. 1a). ΒΒRF2Δ folds into a compact globular domain containing 10 helices and 6 β-strands (Fig. 1b). β1 and β2, connected in-line by a short bulged loop, lie antiparallel to the long β4. β2–β6 form a central sheet sandwiched by two piles of helices. Τhe upper pile contains ɑ1 and bundled ɑ3–ɑ6, and the lower pile contains ɑ2, ɑ7–ɑ9, and a short 3_10_ helix η1.

**Table 1:** Crystallographic data collection and refinement.

	BBRF2Δ	BBRF2Δ-BSRF1Δ complex
*Data collection*		
Space group	P2_1_2_1_2	P2_1_
Cell dimensions		
a, b, c (Å)	73.318, 61.571, 58.109	75.78, 161.85, 97.86
α, β, γ (°)	90, 90, 90	90, 100.8, 90
Wavelength (Å)	0.97915	0.97853
Resolution (Å)^a^	47.15–1.6 (1.69–1.6)	48.07–3.09 (3.28–3.09)
Total reflections	248,156 (39,396)	145,743 (21,580)
Unique reflections	66,863 (10,656)	42,196 (6720)
*R*_*sym*_	0.072 (0.575)	0.082 (0.495)
*I/σ(I)*	13.39 (3.98)	13.27 (2.23)
Completeness (%)	99.1 (97.5)	99.4 (98.3)
Redundancy	3.71 (3.7)	3.45 (3.21)
Refinement		
*R*_work_*/R*_free_	0.166/0.189	0.217/0.27
No. of atoms		
Protein	2153	16,133
Ligand/ion	14	30
Water	278	28
B-factors		
Protein	23.15	75.67
Ligand/ion	56.60	65.38
Water	37.23	44.86
*R.m.s*. deviations		
Bond lengths (Å)	0.009	0.009
Bond angles (°)	1.11	1.10
Ramachandran		
Favoured (%)	98.5	92.0
Outliers (%)	0	1.1

^a^Numbers in parentheses are values from the highest resolution shell.

Arg27 on the bulged loop between β1 and β2 forms salt bridges with Asp85 and Glu122, two highly conserved residues within herpesviruses (Fig. 1c, Supplementary Fig. 2). Glu122 further stabilizes the conformation of the bulged loop through two hydrogen bonds with the main chain nitrogen atoms of Leu28 and Val29, and Asp85 forms a salt bridge with His253 from η1. In addition, these residues and two deeply buried water molecules establish a comprehensive hydrogen bonding network with Asn123 and ambient main chain atoms. All of these interactions contribute to a prominent hydrophilic core at the center of BBRF2 (Fig. 1c). According to the Dali server^21^, the fold of BBRF2 does not resemble that of any existing protein structure. Thus, BBRF2 represents a novel protein domain, and we named it herpesvirus tegument fold 1 (HTF1).

### Overview of the BBRF2-BSRF1 complex structure

**Fig. 2:**
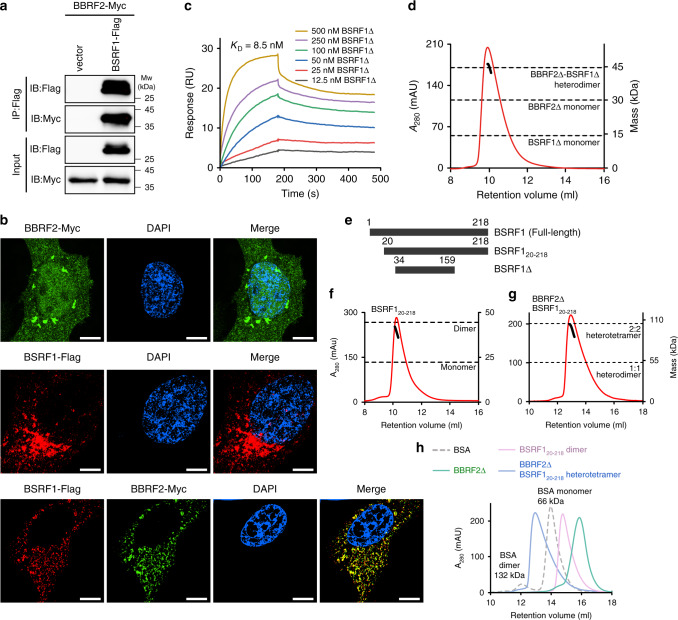
BBRF2 interacts with BSRF1. **a** Co-immunoprecipitation of BBRF2-Myc and BSRF1-Flag in HEK293T cells. The Flag vector was used as a negative control. IB immunoblotting; Input, whole cell lysis. Source data are provided as a Source Data file. **b** Confocal immunofluorescence imaging of BBRF2 (green) and BSRF1 (red) in HeLa cells. Cell nuclei were counterstained with DAPI (blue). Scale bars, 5 μm. Each experiment was repeated three times independently with similar results. **c** The binding affinity of BBRF2Δ with BSRF1Δ measured by Biacore. His-tagged BBRF2Δ (2.5 μg ml^−1^) was immobilized on the NTA chip and the association signals of BSRF1Δ at different concentrations (12.5−500 nM) monitored. Source data are provided as a Source Data file. **d** SEC-RALS analysis showing BBRF2Δ and BSRF1Δ form heterodimers in solution, analyzed on a Superdex 75 SEC column. Calculated molecular masses at the absorption peak of 280 nm are plotted in black. mAU, milli-absorption units. **e** The constructs of full-length BSRF1, BSRF1_20−218_, and BSRF1Δ. **f**, **g** SEC-RALS analysis showing that BSRF1_20−218_ is dimeric in solution (Superdex 75), and BBRF2Δ and BSRF1_20−218_ form heterotetramer in solution (Superdex 200). **h** SEC analysis of the BBRF2Δ-BSRF1_20−218_ complex (blue), BSRF1_20−218_ dimer (pink) and BBRF2Δ (green), with BSA (grey dashed line) as a reference (Superdex 200).

Little was known about the function of BBRF2 at the time we began to study it. We started our exploration by seeking the potential interaction partners of BBRF2 in HEK293 M81 cells, a modified HEK293T cell line with stable infection of recombinant EBV^22^. A GST-pulldown assay was carried out using GST-tagged BBRF2 and HEK293 M81 cell lysate. The resulting candidates were identified by mass spectrometry. Three EBV proteins associated with BBRF2, including the major capsid protein (MCP, or BcLF1) and two tegument proteins, BSRF1 and BPLF1 (Supplementary Fig. 3a). BPLF1 has been reported to have deubiquitinase activity^23^, and its HSV-1 homologue pUL36 has been shown to be attached to the viral capsid via a C-terminal helix^24^. We had a particular interest in BSRF1 because its function was relatively unclear compared to MCP and BPLF1. We confirmed the interaction between BBRF2 and BSRF1 by co-immunoprecipitation (co-IP). When co-expressed in HEK293T cells, Myc-tagged BBRF2 was pulled down by Flag-tagged BSRF1 (Fig. 2a). Both GST-tagged full-length BBRF2 and BBRF2Δ pulled down Flag-tagged BSRF1, suggesting that the N-terminal truncation did not perturb binding with BSRF1 (Supplementary Fig. 3b). In HeLa cells, co-transfected Myc-tagged BBRF2 and Flag-tagged BSRF1 colocalized (Fig. 2b). These results are consistent with the previously reported association between HSV-1 pUL7 and pUL51^12^, and a recent study of BSRF1 interaction partners in HEK293T cells with an incorporated EBV genome^14^. Therefore, these two tegument proteins clearly form a functional complex in a conserved manner across Herpesviridae and we focused on studying the structure of the BBRF2-BSRF1 complex to understand the molecular basis of their association.

Full-length BSRF1 (residues 1–218) was insoluble. We performed construct optimization and obtained a soluble truncated version containing residues 34–159 (termed BSRF1Δ). Surface plasmon resonance (SPR) analysis revealed that BSRF1Δ bound BBRF2Δ with a dissociation constant (K_D_) in the nanomolar range (Fig. 2c). We incubated purified BSRF1Δ and BBRF2Δ together and analyzed the mixture by size-exclusion chromatography coupled with right angle light scattering (SEC-RALS). The two proteins co-eluted in a discrete peak, and the derived molecular mass suggested that they form a stable 1:1 heterodimer in solution (Fig. 2d). Interestingly, a near-full-length version of BSRF1 with intact C-terminus (BSRF1_20−218_) dimerized in solution (Fig. 2e and f), and the apparent molecular mass of the BBRF2Δ-BSRF1_20−218_ complex corresponded to that of a 2:2 heterotetramer (Fig. 2g and h), suggesting that the BBRF2-BSRF1 complex oligomerizes in cells. Initial crystallization of the BBRF2Δ-BSRF1Δ complex did not yield crystals, and degradation of BSRF1Δ was observed. The BBRF2Δ-BSRF1Δ complex was subjected to mild proteolytic conditions and analyzed by mass spectrometry. The degradation occurred at both the N- and C-termini (Supplementary Fig. 4a). To facilitate crystallization, we performed a limited proteolysis test in the crystallization solution using α-chymotrypsin, which resulted in a stable degraded version of BSRF1 (Supplementary Fig. 4b). Finally, we obtained crystals of the BBRF2Δ-BSRF1Δ complex and solved its structure at 3.1 Å. The final model was refined to an *R*_free_ of 0.27 (Table 1). In the asymmetric unit, BBRF2Δ and BSRF1Δ formed a 6:6 heterododecamer (Supplementary Fig. 5a). BSRF1Δ forms two ɑ-helices with two loops at the N- and C-termini. Using the PISA server^25^, we found a predominantly large interface area of ~1300 Å^2^ between BBRF2Δ-1 and BSRF1Δ-1, which we think mediates the constitutive heterodimer observed in solution (Supplementary Fig. 5b). Three constitutive heterodimers are associated in a symmetric manner to build up a triangular 3:3 heterohexamer (Supplementary Fig. 5a). For example, one BSRF1Δ (BSRF1Δ-1) molecule crosses another BSRF1Δ (BSRF1Δ-2) by 60° and contacts both molecules of the corresponding constitutive dimer (BSRF1Δ-2 and BBRF2Δ-2). In this contact, a disulfide bond between Cys95 of BSRF1Δ-1 and Cys217 of BBRF2Δ-2, together with hydrophobic interactions and a hydrogen bond, stabilize the 3:3 heterohexameric assembly (Supplementary Fig. 5c). Mutation of BSRF1 Cys95 to serine did not perturb the BBRF2Δ-BSRF1Δ heterodimer in solution, suggesting that this disulfide bond is not responsible for the association between BBRF2Δ and BSRF1Δ in the constitutive heterodimer (Supplementary Fig. 6a). Two copies of the triangular 3:3 BBRF2Δ-BSRF1Δ heterohexamer stack face-to-face, resulting in a two-layer 6:6 heterododecamer in the asymmetric unit (Supplementary Fig. 5a and 6b). Each constitutive heterodimer associates with two heterodimers from the other layer via salt bridges and hydrogen bonds (Supplementary Fig. 5a).

### Structural basis of the interaction between BBRF2 and BSRF1

**Fig. 3:**
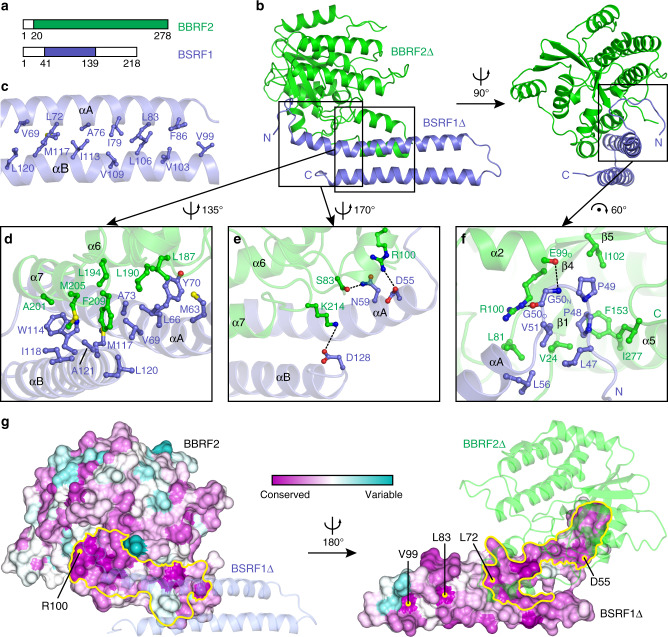
Details of the BBRF2Δ-BSRF1Δ interface. **a** Schematic showing the discernible residues of BBRF2Δ and BSRF1Δ in the BBRF2Δ-BSRF1Δ crystal complex (coloured region) compared to full-length BBRF2 and BSRF1. Colour as in Fig. 3b. **b** Two views of the constitutive heterodimer. Colour as in **a**. The N- and C-termini of BSRF1Δ are specified. **c** Hydrophobic interactions between the two antiparallel ɑ-helices of BSRF1Δ. **d–f** Detailed residue contact at the BBRF2Δ-BSRF1Δ interface. The hydrophobic interface (**d**), polar interface (**e**), and BSRF1 ΔN-terminal loop mediated interface (**f**) are shown. Secondary structural elements are labelled. Involved residues are coloured as the molecule to which they belong. **g** Surface conservation plot of BBRF2Δ (left) and BSRF1Δ (right). Associated BSRF1Δ (left) and BBRF2Δ (right) are shown with transparency for clarity. The interface of the BBRF2Δ-BSRF1Δ heterodimer is outlined in yellow.

In the BBRF2Δ-BSRF1Δ constitutive heterodimer, residues 20–278 of BBRF2Δ are discernable, including 174–178, which are missing from its solo structure. BBRF2Δ did not exhibit a major conformational change before and after complexing with BSRF1Δ (Supplementary Fig. 7a). The BBRF2Δ N-terminal extension that was not resolved in the complex structure would need to be relocated because it occupies the docking site for BSRF1Δ (Supplementary Fig. 7b). Residues 41–139 of BSRF1Δ are resolved in the model (Fig. 3a). The two antiparallel aligned ɑ-helices (ɑA and ɑB), which are connected by a short loop, are closely associated via hydrophobic interactions (Fig. 3b and c). One out of six BSRF1Δ molecules had a longer C-terminus resolved in the electron density, forming a short ɑ-helix ɑC (Supplementary Fig. 7c). This short ɑ-helix does not contribute to the intermolecular interaction. Search in the Dali server resulted in many proteins that partially resemble BSRF1Δ in folding, including those involved in membrane shaping and intracellular trafficking (Supplementary Fig. 7d). Given this similarity, we were curious whether BSRF1Δ can directly interact with membranes. Membrane anchoring of BSRF1 may be dependent on palmitoylation at an N-terminal cysteine, which is highly conserved in Herpesviridae (Supplementary Fig. 2). The corresponding cysteine was reported to mediate the membrane association of HSV-1 pUL51^26^. The BSRF1_20–218_ construct lacking this cysteine displayed a diffuse subcellular localization pattern in immunofluorescent analysis (Supplementary Fig. 8a). We performed a liposome floatation assay using various lipid compositions to mimic the Golgi membrane, endoplasmic reticulum (ER), and plasma membrane. Alone or complexed with full-length BBRF2, BSRF1_20–218_ did not exhibit a strong tendency for membrane binding (Supplementary Fig. 8b). These results reflect the importance of the conserved N-terminal cysteine in mediating membrane associations. However, as the two antiparallel ɑ-helices fold is common, it is difficult to derive decisive clues about the cellular function of BSRF1 directly from its structure.

The antiparallel ɑ6 and ɑ7 of BBRF2Δ join at the middle of ɑA and ɑB of BSRF1Δ, forming a tight heterogeneous four-helix bundle (Fig. 3d). A massive hydrophobic network stabilizes this helical bundle and constitutes the major interface of the BBRF2Δ-BSRF1Δ complex. Two bulky residues on ɑ7 of BBRF2Δ, Met205 and Phe209, wedge into the hydrophobic niche of BSRF1Δ, and hydrophobic residues from ɑ6 of BBRF2Δ add to this cluster (Fig. 3d, Supplementary Fig. 9a). The proximal end of BSRF1Δ creates a hydrophilic interface with BBRF2Δ via two intermolecular salt bridges and a hydrogen bond (Fig. 3e). In addition, the N-terminal loop (N-loop) of BSRF1Δ winds toward the central β-sheet of BBRF2Δ, and a bulged 47-LPPGV-51 motif from the N-loop lies inside a hydrophobic groove (Fig. 3f).

Although BBRF2 and BSRF1 both have counterparts in all three herpesvirus subfamilies, these homologues exhibit substantial sequence divergence (Supplementary Fig. 2). BBRF2 and BSRF1 share only 12.1% and 13.5% sequence similarity to their putative HSV-1 homologues pUL7 and pUL51, respectively. We generated surface conservation plots for the BBRF2Δ and BSRF1Δ structures based on the sequence alignment of 10 herpesviruses from three herpesvirus subfamilies. We found that the BBRF2Δ-BSRF1Δ interface is relatively conserved (Fig. 3g), particularly for the hydrophobic cluster and the intermolecular salt bridge between Arg100 of BBRF2 and Asp55 of BSRF1 (Supplementary Fig. 2). Other highly conserved residues are mainly involved in intramolecular interactions. For example, highly conserved Leu72, Leu83, and Val99 mediate the association between ɑA and ɑB of BSRF1 (Fig. 3c and g). Intriguingly, we noticed that the symmetrical interface of the triangular BBRF2Δ-BSRF1Δ 3:3 heterohexamer was considerably conserved in herpesviruses. Corresponding residues include Val112 and Val122 from BSRF1Δ, which are responsible for the intermolecular hydrophobic interactions, as well as Lys105 from BSRF1Δ, which forms hydrogen bonds with neighbouring BBRF2Δ (Supplementary Figs. 2 and 9b). Such conservation implies that this heterohexamer interface may have functional relevance in the life cycle of herpesvirus.

### Characterization of the BBRF2-BSRF1 interface

**Fig. 4:**
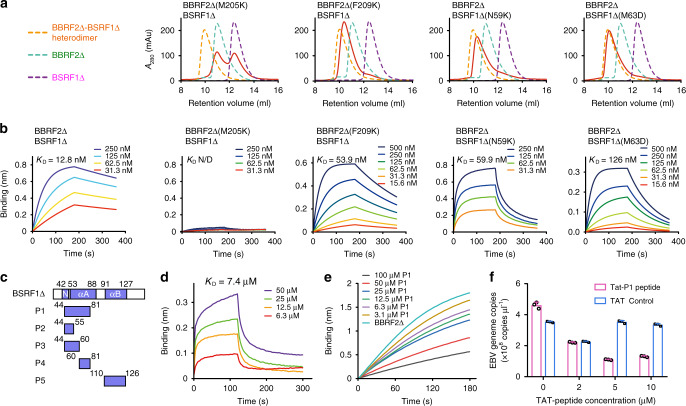
Characterization of the BBRF2-BSRF1 heterodimeric interface. **a** SEC analysis of BBRF2Δ-BSRF1Δ interface mutants (Superdex 75). mAU milli-absorption units. The elution peaks are shown as dashed lines of BBRF2Δ-BSRF1Δ heterodimer (orange), BBRF2Δ monomer (green), BSRF1Δ monomer (purple) are overlaid with each sample (red) as references. **b** The binding affinity of BBRF2Δ (wild-type or mutant) with BSRF1Δ (wild-type or mutant) measured by BLI. BSRF1Δ (wild-type or mutant) at different concentrations was exposed to 10 μg ml^−1^ His-tagged BBRF2Δ (wild-type or mutant). Source data are provided as a Source Data file. **c** Schematic showing the designation of the five BSRF1Δ-derived peptides. N denotes the N-loop. **d** Binding affinity of peptide 1 (P1) and BBRF2Δ measured by BLI. P1 at different concentrations was exposed to 10 μg ml^−1^ His-tagged BBRF2Δ. Source data are provided as a Source Data file. **e** P1 competes with BSRF1Δ in binding to BBRF2Δ. 200 nM BBRF2Δ mixed with different concentrations of P1 was exposed to 10 μg ml^−1^ immobilized BSRF1Δ. Source data are provided as a Source Data file. **f** TAT-P1 reduces the numbers of EBV genome copies in a concentration-dependent manner. Error bar indicates s.d. (*n* = 3). Source data are provided as a Source Data file.

We performed mutagenesis analysis to verify the BBRF2-BSRF1 interface. The two bulky hydrophobic residues of BBRF2 on the interface, Met205 and Phe209, were individually mutated to a charged lysine. In size-exclusion chromatography (SEC), BBRF2Δ(M205K) did not form a complex with BSRF1Δ, whereas BBRF2Δ(F209K) maintained the binding for BSRF1Δ (Fig. 4a). Mutations in two nonconserved residues of BSRF1, Asn59, which mediates an intermolecular hydrogen bond, and Met63 in the outer shell of the hydrophobic network of the heterogeneous four-helix bundle, did not perturb the formation of BBRF2Δ-BSRF1Δ heterodimer (Fig. 4a). We also performed SEC with BBRF2Δ and BSRF1Δ mutants regarding other assembly interfaces of the crystallographic 6:6 heterododecamer shown in Supplementary Figs. 5. None of these mutants affected BBRF2Δ-BSRF1Δ heterodimerization or BBRF2Δ-BSRF1_20−218_ heterotetramerization (Supplementary Figs. 10a and b). Next, we performed biolayer interferometry (BLI) to compare the binding affinities of these mutants for their partners; the results were consistent with the SEC analysis. BBRF2Δ(M205K) did not bind BSRF1Δ. BBRF2Δ(F209K), BSRF1Δ(N59K), and BSRF1Δ(M63D) exhibited moderately reduced binding affinity for BSRF1Δ or BBRF2Δ (Fig. 4b). Thus, only the mutation of BBRF2 Met205 at the center of the heterogeneous four-helix bundle could efficiently abolish the tight interaction between BBRF2Δ and BSRF1Δ. These observations confirmed that the extensive hydrophobic interface between BBRF2Δ and BSRF1Δ mediates the formation of the constitutive heterodimer.

As the interaction between BBRF2 and BSRF1 (or their homologues) has been reported to play a role in assembly during the life cycle of several pathogenic human herpesviruses^12,14,19^, targeting this interface could be a potential clinical strategy against EBV. In light of the verified structural details of the BBRF2Δ-BSRF1Δ interface, we designed five BSRF1-derived peptides (P1–P5), covering the BBRF2-associating sites at the N-loop, ɑA, or ɑB of BSRF1 (Fig. 4c, Supplementary Fig. 11a). According to the BLI analysis, only the longest P1, which covers both the N-loop and ɑA and corresponds to the pUL7 interaction domain as shown for HSV-1 pUL51^20^, presented substantial binding to BBRF2Δ with a K_D_ of 7.4 μM (Fig. 4d, Supplementary Fig. 11b). This means that a single structural element of BSRF1 is insufficient to confer the stable interaction with BBRF2. Next, we examined whether P1 is able to compete with wild-type BSRF1Δ in the BBRF2Δ-BSRF1Δ complex. As indicated by the BLI assays, addition of P1 undermined the association between BBRF2Δ and immobilized BSRF1Δ in a concentration-dependent manner (Fig. 4e), suggesting that P1 can compete with BSRF1 in binding BBRF2Δ. Given this positive result, we investigated the potential cellular effects of P1 in CNE2-EBV cells^27^, which are human nasopharyngeal carcinoma cells with sustained EBV infection. To facilitate the uptake of P1 by cells, we fused a TAT sequence and a quadruple-glycine linker to the N-terminal of P1 (TAT-P1). When supplied to cultured CNE2-EBV cells, TAT-P1 reduced the number of viral genome copies in a concentration-dependent manner. At a concentration of 5 μΜ, TAT-P1 was able to decrease the number of EBV genome copies by 75%, although this effect was not further promoted at a higher concentration of TAT-P1 (Fig. 4f). These results indicate that disrupting the BBRF2-BSRF1 interaction could be an effective way of controlling EBV virion production.

### Role of the BBRF2-BSRF1 complex in viral envelopment

**Fig. 5:**
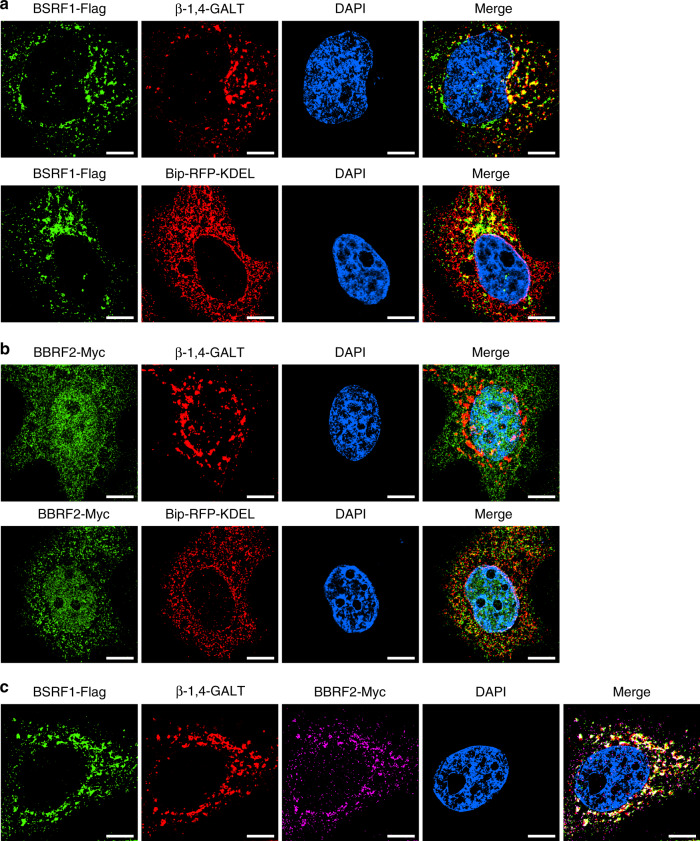
Subcellular localization of BBRF2 and BSRF1. **a**–**c** The subcellular localization of exogenous BSRF1 (**a**), BBRF2 (**b**), and co-expressed BSRF1 and BBRF2 (**c**) analyzed by confocal immunofluorescence. β-1,4-GALT and Bip-RFP-KDEL indicate the location of the Golgi apparatus and ER, respectively. Scale bar, 5 μm. Each experiment was repeated three times independently with similar results.

A previous study demonstrated that knockout of BBRF2 significantly reduces the infectivity of progeny virus, and BBRF2 protects BSRF1 from degradation in EBV-infected Akata cells^15^. Depletion of BSRF1 does not affect EBV lytic replication, but its knockdown in B95-8 cells reduces progeny production^14^. Similar results have been reported for BSRF1 homologues in various herpesviruses^12,17^. These results imply that the BBRF2-BSRF1 complex plays a role in the assembly of EBV. Thus, we evaluated the subcellular localization of BBRF2 and BSRF1. As revealed by immunofluorescence in HeLa cells, Flag-tagged BSRF1 mainly localized at the Golgi apparatus, and Myc-tagged BBRF2 did not associate specifically with the Golgi apparatus or ER (Fig. 5a and b). When co-transfected into HeLa cells, BBRF2 and BSRF1, likely in the complexed form, accumulated at the Golgi apparatus (Fig. 5c). These observations are consistent with previous studies on BBRF2 and BSRF1, as well as their homologues in other herpesviruses^12,14^.

**Fig. 6:**
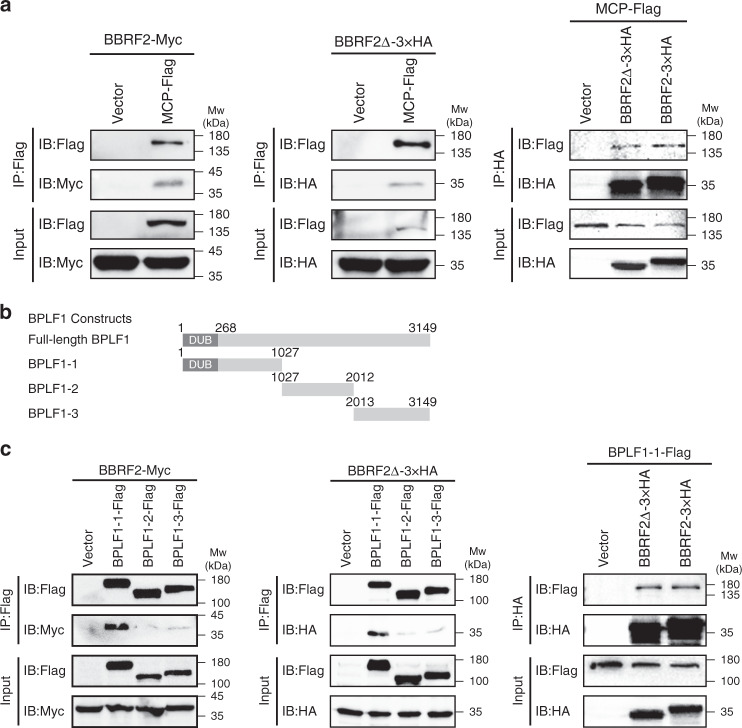
BBRF2 binds MCP and capsid-associated BPLF1. **a** Reciprocal co-IP of EBV MCP and BBRF2/BBRF2Δ in HEK293T cells. Empty vectors were used as negative controls. IB immunoblotting; Input, whole cell lysis. Source data are provided as a Source Data file. **b** Truncated versions of BPLF1. Residue numbers are indicated at the borders. DUB deubiquitination (DUB) domain. **c** Co-IP of BPLF1 fragments and BBRF2/BBRF2Δ. The association between BPLF1-1 and BBRF2/BBRF2Δ was further verified by a reciprocal co-IP test. Source data are provided as a Source Data file. Each experiment was repeated three times independently with similar results.

As implied by the GST-pulldown assay, BBRF2 associates with MCP and BPLF1 (Supplementary Fig. 3a). We confirmed these associations by co-IP assays in HEK293T cells. MCP co-precipitated with both full-length BBRF2 and BBRF2Δ (Fig. 6a). BPLF1 is a large tegument protein containing more than 3,000 amino acid residues. We generated three truncated BPLF1 constructs of similar sizes and applied them to co-IP assays (Fig. 6b). The N-terminal part (residues 1–1027) of BPLF1, which contains a deubiquitination (DUB) domain conserved in Herpesviridae^28^, co-precipitated with full-length BBRF2 and BBRF2Δ (Fig. 6c). These results were verified by reciprocal co-IP tests in which BBRF2 and BBRF2Δ co-precipitated with MCP and BPLF1_1–1027_ (Fig. 6a and c).

**Fig. 7:**
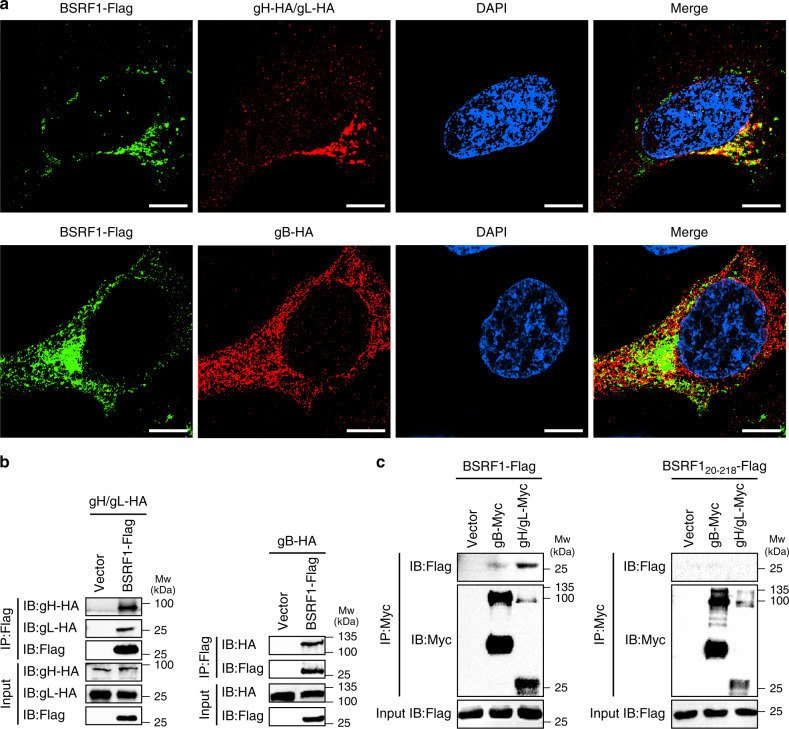
BSRF1 associates with EBV glycoproteins. **a** BSRF1 co-localizes with EBV glycoproteins. Green, BSRF1-Flag; red, glycoproteins. Nuclei were stained with DAPI (blue). Scale bar, 5 μm. **b** Flag-tagged BSRF1 co-immunoprecipitated with HA-tagged EBV glycoproteins in HEK293T cells. Source data are provided as a Source Data file. **c** Myc-tagged EBV glycoproteins co-immunoprecipitated with Flag-tagged BSRF1 and BSRF1_20−218_. Source data are provided as a Source Data file. Each experiment was repeated three times independently with similar results.

Several tegument proteins are functionally associated with glycoproteins. For example, direct interactions between pUL11/pUL16 and glycoprotein gE, as well as between pUL37 and glycoprotein gK, have been reported for HSV-1^29,30^. Recently, pUL51 was shown to interact with glycoprotein gE^31^. These clues prompted us to determine whether BBRF2 or BSRF1 binds to EBV glycoproteins. Immunofluorescence indicated that BSRF1 co-localizes with the EBV glycoprotein gH-gL complex in the juxtanuclear compartment of HeLa cells after co-transfection (Fig. 7a), whereas colocalization between BBRF2 and the glycoproteins was not evident in the same experiment (Supplementary Fig. 12). When exogenously overexpressed, the gH-gL complex resides mainly in the Golgi apparatus and gB in the ER^32^. This difference in organelle residence may account for the observation that BSRF1 exhibited a more prominent colocalization with gH-gL than gB (Fig. 7a). Under physiological conditions, gB may exist in the Golgi apparatus at certain stages of the EBV life cycle, as its homologues in several other herpesviruses are reported to have Golgi distribution^33–35^. To further validate the association between BSRF1 and glycoproteins, we performed co-IP assays. BSRF1 co-precipitated with EBV gH-gL and gB (Fig. 7b). Reciprocally, EBV gH-gL and gB co-precipitated with BSRF1, but not the truncated version BSRF1_20–218_ (Fig. 7c). HSV-1 gH and gB did not co-precipitate with BSRF1 (Supplementary Fig. 13a). To examine the possibility of nonspecific interactions between BSRF1 and host Golgi proteins, we tested several Golgi proteins in the co-IP assay: GOLGA5, which is spread throughout the Golgi apparatus; GOLGA1, which resides in the *trans* face; GM130, which resides in the *cis* face; and GORASP2, which is found in the cisternae. BSRF1 co-precipitated only with GOLGA5 (Supplementary Fig. 13b). These results suggest that BSRF1 associates with gH-gL and gB, and the 19 N-terminal residues of BSRF1 are responsible for this association.

## Discussion

**Fig. 8:**
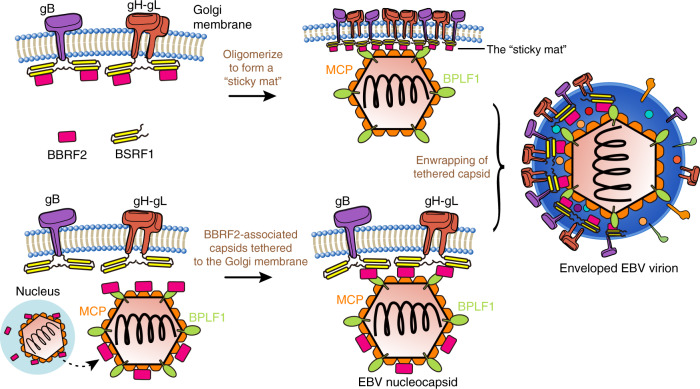
Model of BBRF2-BSRF1 complex-mediated EBV secondary envelopment. Proposed working model for the BBRF2-BSRF1 complex during secondary envelopment of EBV. BSRF1 and BBRF2 form stable heterodimers at the cytoplasmic site of the Golgi apparatus, and are associated with glycoproteins. Next, the BBRF2-BSRF1 complexes oligomerize, creating a “stick mat”, which tethers EBV nucleocapsids to the Golgi apparatus and eventually facilitates the secondary envelopment process. Alternatively, the EBV capsid may bind BBRF2 in the cell nucleus prior to entering the cytoplasm. The BBRF2-associated capsid then binds Golgi-localized BSRF1.

Research on the tegument protein interaction network and their role in secondary envelopment has mainly converged on α-herpesviruses such as HSV-1^9^, but few on EBV. In this study, we reported a complex structure of EBV tegument proteins BBRF2 and BSRF1 that reveals a conserved mode of crosstalk between tegument proteins in all three families of herpesviruses. The results of our biochemical and cell-based experiments, together with previous studies on BBRF2/BSRF1 homologues in other herpesviruses, demonstrated the importance of the BBRF2-BSRF1 complex in the assembly of EBV virions, with strong implications for secondary envelopment^12,17^. Based on these structural and functional implications, we propose a mechanism for how the BBRF2-BSRF1 complex facilitates secondary envelopment. Briefly, when expressed in EBV-infected cells, BSRF1 and BBRF2 form a stable complex and are enriched at the Golgi apparatus. As BBRF2 associates with MCP and BPLF1 from EBV nucleocapsids, the accumulated BBRF2-BSRF1 complex may serve as a molecular sticker at the Golgi apparatus that tethers the juxtaposing de-enveloped capsids exported from the cell nucleus to the glycoprotein-embedded Golgi membrane, eventually promoting the membrane to enwrap the tethered capsids (Fig. 8).

A first key point of this model is how the BBRF2-BSRF1 complex is attached to the Golgi apparatus. Immunofluorescence studies, both ours and others’, have confirmed the distribution of BSRF1 at the Golgi apparatus^14^. As discussed above, anchoring of BSRF1 to the Golgi membrane is most likely dependent on palmitoylation at the conserved N-terminal cysteine, as in HSV-1 pUL51^26^, which is supported by the observation that BSRF1_20–218_ lacking the palmitoylatable cysteine failed to localize to the Golgi apparatus (Supplementary Fig. 8a). pUL51 has been reported to localize to the cytoplasmic surface of the Golgi membrane when overexpressed alone, whereas it resides mainly inside the cytoplasmic vesicles or the viral envelope in HSV-1-infected cells^26^. This observation sheds light on our model: the Golgi-localized BSRF1 may initially remain on the cytoplasmic side, and its internalization with nucleocapsids or other viral proteins is the consequence of secondary envelopment. For HSV-1, the recruitment of pUL7 to the cytoplasmic membrane requires pUL51 or other partners^12^. The subcellular localization patterns of BBRF2 alone or with co-expression of BSRF1 that have been observed by other groups^15^ and in our study suggest a similar mechanism as in HSV-1 in which BBRF2 can be recruited to the Golgi apparatus by BSRF1 (Figs. 2b and 5c). The expression levels of pUL7 and pUL51 rely on the presence of each other for HSV-1^17^. If this is the case for BBRF2 and BSRF1 in EBV, it is very likely that in the late lytic cycle of EBV, the two proteins form stable complexes on the cytoplasmic site of the Golgi apparatus before the nucleocapsids are translocated to the cytoplasm.

The next question is how the BBRF2-BSRF1 complex efficiently tether EBV nucleocapsids. We found that BBRF2 associates with MCP and BPLF1 (Fig. 6a and c). MCP occupies the majority of the surface area of naked capsids^24,36^. BPLF1 is the largest tegument protein (~340 KDa), with functional implications in promoting viral DNA replication by disrupting the activity of cullin-RING ligases^37^. The BPLF1 homologues in HSV-1 and pseudorabies virus (pUL36 or VP1/2) have been reported to mediate the retrograde trafficking of viral capsids by recruiting host motor proteins^38,39^. A major feature of pUL36 is that it constitutes a so-called capsid vertex-specific component with two capsid proteins pUL17 and pUL25^40^. As indicated by cryo-EM studies, the C-terminal ɑ-helix of pUL36 regularly forms a bundle that is integrated into the capsid with ɑ-helices from pUL17 and pUL25, but the rest of pUL36 is absent from the electron density^24,41^. These findings have confirmed previous conclusions regarding the role of pUL36 as a flexible scaffold linking capsid and tegument structures^9,42^. Therefore, it is tempting to speculate that the association between BBRF2 and MCP, or between BBRF2 and BPLF1, or both, promotes the tethering of Golgi-associated BBRF2-BSRF1 complex to viral capsids. The current data do not substantiate direct interactions between BSRF1 and EBV glycoproteins, or between BBRF2 and MCP/BPLF1. Further investigation is needed to understand the molecular basis of these associations. In addition, given the nuclear localization of BBRF2 when exogenously expressed alone, it is also possible that the viral capsids already have BBRF2 bound to the surface before they enter the cytoplasm and attach to the Golgi apparatus (Fig. 8).

In SEC-RALS, we found that near-full-length BSRF1 exhibits a tendency for oligomerization, both alone and in complex with BBRF2Δ (Fig. 2f and g). As the BSRF1Δ is monomeric, the putative oligomerization is likely to be mediated by the C-terminal region of BSRF1 that is not present in the crystal structure. This finding is in accordance with the reported oligomerization of BSRF1 homologues in other herpesviruses^43–45^. Whether the conserved hydrophobic interface (especially the intermolecular disulfide bond) observed with the crystallographic 3:3 heterohexamer (Supplementary Fig. 5c and Supplementary Fig. 9b) contributes to the in-cell oligomerization of BBRF2-BSRF1 complex requires further investigation. Oligomerized BBRF2-BSRF1 complex may exist at the Golgi membrane in the late stage of the EBV lytic cycle, and create a sticky mat-like surface to provide enough contact area for the nucleocapsids. As we proved that BSRF1 associates with EBV glycoproteins (Fig. 7b and c), it is possible that oligomerized BBRF2-BSRF1 complexes facilitate the clustering of attached glycoproteins. The tethering between the BBRF2-BSRF1 sticky mat and viral capsid, perhaps at just one side of the icosahedral shell, may mark the initiation of the membrane enwrapping process and be sustained even after envelopment. This speculation is consistent with the cryo-EM images of mature virions showing the asymmetric placement of herpesvirus capsid inside the envelope, where the thicker distal pole is dominated by the tegument- and glycoprotein-rich membrane^46–48^.

Overall, the BBRF2-BSRF1 complex may create a tethering-permissive environment that facilitates the secondary envelopment of EBV. Other tegument protein complexes with potential functions in promoting secondary envelopment include HSV-1 pUL11-pUL16-pUL21 and pUL36-pUL37^9,49^. Unlike the direct association between BBRF2 and BSRF1, formation of the pUL11-pUL16-pUL21 tripartite complex is dependent on the participation of glycoprotein gE, which is needed for efficient cell-to-cell spread of virions^30,50,51^. pUL36 seems to be stably anchored on viral capsids before recruiting pUL37^24^. These HSV-1 proteins have putative homologues in EBV, but whether these EBV tegument proteins form similar functional complex is unknown. If yes, they may have some redundancy with the BBRF2-BSRF1 complex. On the other hand, given that knockdown of either BBRF2 or BSRF1 reduces the number of viral genome copies in EBV-infected cells^14,15^, the BBRF2-BSRF1 complex and other pUL11-pUL16-pUL21- or pUL36-pUL37-like tegument protein complexes of EBV, if present, may conduct different critical processes in the maturation and egress of EBV. Finally, the unique fold of BBRF2 makes it a potential specific drug target against EBV. As the P1 peptide has shown an inhibitory effect on EBV genome copies at low micromolar levels (Fig. 4f), targeting the BBRF2-BSRF1 complex with optimized BSRF1-derived peptides may be a novel strategy for treating EBV infection and EBV-related human cancer.

## Methods

### Protein expression and purification

The BBRF2 and BSRF1 cDNAs were amplified from the human herpesvirus 4 strain M81 genome. The cDNAs for full-length BBRF2 and BBRF2_17–278_ were cloned into a modified pET28 vector (pSKB) and expressed as a fusion protein with an N-terminal 6×His-tag followed by a PreScission cleavage site in *E*. *coli* Rosetta (DE3) cells (primer information in Supplementary Table 1). All mutants were generated by site-directed mutagenesis and confirmed by sequencing (primer information in Supplementary Table 2). Transformed bacteria were cultured at 37 °C in Terrific Broth (TB) medium and induced by the addition of 100 μM isopropyl-1-thio-β-D-thiogalactopyranoside (IPTG) at an optical density of 0.6. After induction, cells were grown overnight (~16–18 h) at 18 °C and collected by centrifugation. Collected cells were lysed in ice-cold buffer containing 20 mM HEPES pH 7.0, 600 mM NaCl, 10% glycerol, 30 mM imidazole, 1 μM DNase I, 1 mM phenylmethanesulfonylfluoride (PMSF), and 2 mM β-mercaptoethanol (β-ME) using a cell disruptor (JNBIO), and subjected to centrifugation at 40,000 × *g* for 1 h at 4 °C. The supernatant was filtered and applied to a Ni-NTA (first Ni-NTA) column (GE Healthcare) equilibrated with binding buffer A containing 20 mM HEPES pH 7.0, 600 mM NaCl, 10% glycerol, 30 mM imidazole, 2 mM β-ME. After being washed with binding buffer A, proteins were eluted with an elution buffer containing 20 mM HEPES pH 7.0, 600 mM NaCl, 10% glycerol, 300 mM imidazole, and 2 mM β-ME. Eluted proteins were incubated with 20 μg glutathione S-transferase (GST)-fused PreScission protease (PSP) to remove the His_6_-tag, and dialyzed overnight at 4 °C against binding buffer B containing 20 mM HEPES pH 7.0, 600 mM NaCl, 10% glycerol, and 2 mM β-ME. After dialysis, PSP was removed using a GST column. Proteins were reapplied to a second Ni-NTA column equilibrated with binding buffer B and eluted with binding buffer A. The eluted proteins were subsequently applied to SEC using a HiLoad 16/60 Superdex 75 column (GE Healthcare) equilibrated with buffer C containing 20 mM HEPES pH 7.0, 600 mM NaCl, 10% glycerol, and 1 mM dithiothreitol (DTT). The proteins were eluted in a discrete peak and collected. The selenomethionine (SeMet) derivative of BBRF2 (17−278) was expressed as described previously and purified as native BBRF2 proteins.

The BSRF1_34–159_ cDNA was cloned into pGEX-6p-1 vector. The recombinant proteins were expressed and collected as BBRF2 proteins in a lysis buffer containing 50 mM HEPES pH 7.0, 150 mM NaCl, 1 mM PMSF and 2 mM β-ME. Collected proteins were applied to a GST-column equilibrated with a binding buffer containing 20 mM HEPES pH 7.0, 150 mM NaCl, and 2 mM β-ME, and eluted with a buffer containing 20 mM HEPES pH 7.0, 150 mM NaCl, 10 mM glutathione, and 2 mM β-ME. After being treated with 20 μg PSP, proteins were applied to a GST column to remove the GST-tag and PSP. SEC was carried out following the same protocol as for BBRF2 proteins. The BBRF2_17–278_-BSRF_34–159_ (BBRF2Δ-BSRF1Δ) complex was prepared by mixing and incubating purified BBRF2Δ and BSRF1Δ at 1:1 molar ratio overnight at 4 °C, and purified by SEC using a HiLoad 16/60 Superdex 200 column (GE Healthcare) in buffer C. The BSRF1_20–218_ cDNA was cloned into pSKB. The recombinant proteins were expressed, collected and purified as BBRF2 constructs, with less NaCl (300 mM) and no glycerol for all of the buffers.

### Crystallization

Crystallization experiments were carried out using the hanging drop vapour diffusion method with a mixture of equal volumes of protein (~7 mg ml^−1^) and reservoir solution. The crystals of SeMet BBRF2Δ were grown from 0.09 M NPS (0.03 M NaNO_3_, 0.03 M Na_2_HPO_4_, 0.03 M (NH4)_2_SO_4_), 0.1 M MES/imidazole pH 6.5, 12.5% PEG1000,12.5% PEG 3350, and 12.5% MPD at 4 °C. Crystals of BBRF2Δ-BSRF1Δ complex were grown from 0.1 M magnesium acetate, 0.05 M MES pH 5.6, and 20% MPD after being treated with 1:1000 m/m α-chymotrypsin overnight at 4 °C. The crystals were directly flash frozen in liquid nitrogen.

### Structural determination

X-ray diffraction datasets were collected for BBRF2Δ at beamlines BL17U1 and BL19U1 of the Shanghai Synchrotron Radiation Facility (SSRF)^52^. The dataset for BBRF2Δ-BSRF1Δ complex was collected at beamline BL18U1 of SSRF. The datasets were processed and scaled using the XDS program suite^53^. Initial phases of the BBRF2Δ structure were obtained by the single anomalous dispersion (SAD) method and refined using phenix^54^ from a diffraction dataset of SeMet-substituted BBRF2Δ crystal. The BBRF2Δ-BSRF1Δ complex structure was solved by molecular replacement using Phaser^55^ with the structure of BBRF2Δ as the search model. The model for BSRF1Δ was manually built with COOT^56^. Six copies BBRF2Δ and six copies BSRF1Δ were present in the asymmetric unit. The 6:6 heterododecamer model was refined firstly by Refmac^57^ with NCS restraints and a weighting term of 0.002 until the polypeptide chains were more or less complete, and then by Phenix with translation-libration-screw (TLS) refinement using the BBRF2Δ structure as a reference model. The AutoBuild^58^ programme in the Phenix suite was used to minimize model bias. Structural validation was carried out using MolProbity^59^. Structural illustrations were generated using PyMOL Molecular Graphic Systems (version 0.99, Schrödinger LLC; http://www.pymol.org/) and CCP4mg^60^. X-ray data collection and refinement statistics are listed in Table 1.

### SEC-RALS

A coupled RALS-refractive index detector (Malvern) was connected in-line to SEC using a Superdex 75 or Superdex 200 Increase 10/300 GL column (GE Healthcare) to determine the apparent molecular masses of the applied protein samples. To monitor the association of BBRF2Δ with BSRF1Δ (and corresponding mutants) or BSRF1_20–218_ (wild-type or mutant), 25 μM purified BBRF2Δ (wild-type or mutant) and BSRF1Δ (wild-type or mutant) or BSRF1_20–218_ (wild-type or mutant) were mixed and incubated overnight at 4 °C before being applied to SEC. The entire experiment was performed in buffer C. The RALS data were analyzed by OMNISEC software.

### Cell culture

HEK293T cells were purchased from ATCC (CRL-3216). HEK293 M81 cells, which carry recombinant EBV M81 strain^22^, were a gift from Prof. Dong-Yan Jin (University of Hong Kong). HeLa cells were obtained from Prof. Ran-Yi Liu (Sun-Yet San University Cancer Center). CNE2-EBV cells were obtained Prof. Yi Zeng (Chinese Academy of Medical Sciences). HEK293 M81 cells and HeLa cells were cultured in Dulbecco’s modified essential medium (DMEM) supplemented with 10% fetal bovine serum (FBS, GIBCO) and penicillin/streptomycin. CNE2-EBV cells derived from parental cell lines carrying Akata-EBV-GFP and were cultured in the presence of G418 (500 μg ml^−1^) in RPMI1640 medium (GIBCO) supplemented with 10% FBS and penicillin/streptomycin. All cell lines were free of mycoplasma.

### Antibodies and constructs

The following antibodies were used in this study: ANTI-FLAG^®^ M2 antibody (3165, Sigma,1:1000), DYKDDDK-Tag (14793, CST, 1:1000), Myc-tag monoclonal antibody (2278, CST, 1:1000), HA-tag monoclonal antibody (C29F4/6E2, CST, 1:1000), horseradish peroxidase-conjugated goat-anti-mouse/rabbit secondary antibodies (7076/7071, CST, 1:5000), Alexa Fluor 488/647-conjugated goat-anti-rabbit or mouse (11008/21205, Thermo), and Alexa Fluor 549-conjugated goat-anti-rabbit or mouse (8889/8890, CST). For cell line-based assays, cDNAs for BSRF1, BBRF2, gB (EBV/HSV-1), gH (EBV/HSV-1), gL (EBV/HSV-1), GOLGA1, GM130 (GOLGA2), GOLGA5 and GORASP2, and pcDNA3.1 + (Flag/3×HA-tag), pDORN-pDEST (Myc-tag), pCAGGS (HA-tag), and pcDNA6/myc-His B vectors were used to generate corresponding recombinant plasmids for overexpression. The ER marker (Bip-RFP-KDEL) and the Golgi apparatus marker (β-1,4-galactosyltransferase, β-1,4-GALT) were obtained from Prof. Quentin Liu (Sun Yat-sen University Cancer Center). Primers used for generating the corresponding constructs are listed in Supplementary Table 3.

### Plasmid transfection

Cells were plated at a density of 50–70%. Indicated plasmids were delivered by Lipofectamine 2000 (Invitrogen) according to the manufacturer’s instructions.

### Western blotting

Cells were lysed in RIPA buffer containing Protease Inhibitor Cocktail (Pierce). The protein amount in the lysates was determined using the BCA protein assay kit (Beyotime, China). Samples were normalized to equal amounts of protein, separated by 10–12% SDS-PAGE and transferred to polyvinylidene difluoride (PVDF) membrane (Millipore). The membranes were blocked with 5% skim milk and probed with the primary antibodies. The blots were then incubated with species-specific HRP-conjugated secondary antibodies and visualized by enhanced chemiluminescence (ECL, Tanon).

### GST-pulldown assay

GST-tagged full-length BBRF2 and BBRF2Δ expressed in *E*. *coli* were purified and mixed with glutathione agarose (GE Healthcare) for 4 h at 4 °C. After washing five times with a buffer containing 20 mM HEPES pH 7.0, 150 mM NaCl, and 2 mM β-ME, the resin was incubated with the cell lysates containing bait protein overnight at 4 °C. The resin was washed five times to remove unbound or nonspecific proteins and subjected to SDS-PAGE. The candidate proteins were identified by Western blotting or LC-MS/MS mass spectrometry (Wininnovate Bio).

### Co-immunoprecipitation assay

Cells transfected with recombinant plasmids were lysed in the lysis buffer (25 mM Tris-HCl, pH 7.4, 150 mM NaCl, 1 mM EDTA, 1% NP40, and 5% glycerol) containing 1 mM PMSF and protease inhibitor cocktail (TargetMol), and applied to centrifugation at 15,000 × *g* for 20 min at 4 °C. Cell lysates were subjected to precipitation using 20 μl ANTI-FLAG^®^ M2 Affinity Gel (A2220, Sigma), Anti-c-Myc Tag Affinity Gel (9E10, BioLegend), or Anti-HA Magnetic Beads (88836, Pierce) overnight at 4 °C. The sample was washed five times with the lysis buffer to remove unbound proteins and suspended in 1× SDS loading buffer. The suspended sample was boiled for 5 min at 100 °C before analysis by SDS-PAGE and western blotting with the indicated antibodies.

### Immunofluorescence confocal microscopy

Cells were plated on coverslips for 12–16 h before being transfected accordingly with recombinant plasmids for BBRF2-Myc, BSRF1-Flag, gB-HA, gH/gL-HA, Bip-RFP-KDEL, and/or β-1,4-GALT. After washing twice with PBS, cells were fixed with 4% paraformaldehyde in PBS for 15 min, permeabilized with 0.1% Triton X-100, blocked, and incubated with the indicated antibodies. To stain the cell nuclei, cells were washed with PBS and mounted using DAPI Antifade Fluoromount-GTM (YEASEN) after incubation with Alexa Fluor 488/594/647 antibody. The confocal images were acquired in a structured illumination microscopy (SIM) facility.

### Surface plasmon resonance assay

The surface plasmon resonance (SPR) assay was performed on a Biacore T200 (Biacore). His_6_-tagged BBRF2Δ (dialyzed in a buffer containing 20 mM HEPES pH 7.4, 600 mM NaCl and 1 mM DTT prior to Biacore analysis) was immobilized on an NTA chip (GE 28995043). BSRF1Δ (diluted with the same buffer to achieve concentrations from 12.5 nM to 500 nM) was injected at a speed of 40 μl min^−1^ and passed over the surface of the chip to an equivalent around 300 resonance units (RU). Resonance signals were recorded and analyzed by the Biacore T200 software to derive the dissociation constant (K_D_). The results were presented in a graph using Origin (version 2019, OriginLab).

### Biolayer interferometry assay

The biolayer interferometry (BLI) assay was performed using an eight-channel OctetRED biolayer interferometry system (FortéBio). To measure the interactions between BBRF2Δ and BSRF1Δ variants or peptides P1–P5, His_6_-tagged BBRF2Δ and its variants (10 μg ml^−1^) were immobilized onto NTA biosensor tips (FortéBio) pre-equilibrated with the reaction buffer containing 20 mM HEPES pH 7.0, 600 mM NaCl, 10% glycerol, and 1 mM DTT. BSRF1Δ and its variants or peptides P1–P5 were diluted to between four and eight different concentrations, and analyzed sequentially at each concentration by the tips coated with His_6_-tagged BBRF2Δ (or variants).

To monitor the competitive binding to BBRF2Δ between BSRF1Δ and P1 peptide, BSRF1Δ was biotinylated using the Biotinylation Kit (Genemore) for 30 min at room temperature before being immobilized onto Streptavidin-coated (SA) biosensor tips pre-equilibrated in the reaction buffer. P1 was diluted to different concentrations (100, 50, 25, 12.5, 6.3, and 3.1 μM) in the reaction buffer P1, and individually mixed with 200 nM BBRF2Δ. All experiments were carried out at 25 °C. Each measurement involved an 120 s baseline (with reaction buffer), followed by a 180 s (with protein or peptide) association phase, and a 180 s dissociation phase (with reaction buffer). Raw data were processed using Octet Data Analysis software 11.0 provided by Fortebio to derive the dissociation constant (K_D_). The results were presented in a graph using Origin (version 2019, OriginLab).

### Time-dependent limited proteolysis

Purified BBRF2Δ-BSRF1Δ complex was mixed with α-chymotrypsin (1:1000 m/m) in a buffer containing 20 mM HEPES pH 7.0, 600 mM NaCl, 10% glycerol, and 1 mM DTT at 4 °C. Samples were collected at the indicated time points (0, 1, 4, 8, 12, and 24 h) and the reaction was terminated by adding sample buffer containing 4× SDS before analyzed by 16% Tricine-SDS-PAGE.

### Mass spectrometry

Purified BBRF2Δ-BSRF1Δ complex was treated with α-chymotrypsin (1:1000 m/m) and incubated at 4 °C overnight. Sequences of the proteolytic fragments of BSRF1 were determined by ultra-high performance liquid chromatography/mass spectrometry (UHPLC/MS) using a EASY-nLC 1000 UHPLC system (Thermo Scientific, USA) and a LTQ Velos Pro-Orbitrap Elite mass spectrometer (Thermo Scientific, USA). Data were analyzed by Proteome Discoverer 1.3 software. The molecular masses of BBRF2Δ and BSRF1Δ after digestion were measured by matrix-assisted laser desorption ionization time-of-flight (MALDI-TOF) MS using an UltrafleXtreme MALDI-TOF/TOF instrument (Bruker Daltonics, Germany) equipped with a 355 nm Nd:YAG laser in the positive linear mode. Data were analyzed by FlexAnalysis software.

### Liposome floatation assay

Purified full-length BBRF2, BSRF1_20–218_, and the BBRF2/BSRF1_20-218_ complex were reconstituted into different preformed liposomes. The lipids were mixed and dried under nitrogen gas flow to make a lipid film, which was dried again on the vacuum centrifuge (Concentrator plus, Eppendorf) for 1 h. The lipid film was rehydrated with buffer D containing 20 mM HEPES pH 7.0, 600 mM NaCl, 1 mM DTT to yield a final concentration of 10 mM. The mixture was frozen-thawed 15 times using liquid nitrogen, and homogenized using a mini extruder accompanied by a polycarbonate filter with 200 nm pore size (Avanti Polar Lipids, Inc.) to create large unilamellar liposomes. The plasma membrane liposome mix contained (mol %): 54.5% POPC, 33% PE, 11% DOPS, 1% PI3P, and 0.5% rhodamine-DPPE. The Golgi liposome mix contained (mol %): 52% POPC, 19% PE, 5% DOPS, 15% cholesterol, 8% PI4P, and 1% rhodamine-DPPE. The ER liposome mix contained (mol %): 83.5% POPC, 15% DOPS, and 1.5% rhodamine-DPPE. Protein (6 μM) was incubated with 3 mM liposomes in buffer D for 60 min at room temperature to generate proteoliposomes. To verify the reconstitution efficiency, 50 μl proteoliposomes were mixed with 50 μl of 1.75 M sucrose and overlaid with 80 μl of 0.64 M sucrose and 20 μl of 0.23 M sucrose. After centrifugation in a Beckman TLA 100 rotor at 280,000 × *g* for 70 min at 4 °C, 40 μl aliquots (1/5 fraction each) were collected from the top to the bottom of the sucrose gradient. All fractions were analyzed by 14% SDS-PAGE.

### Peptide synthesis

The peptides were ordered from Sangon Bio (Shanghai, China). The amino acid sequences of the peptides are listed in Supplementary Table 4.

### Peptide transfection assay

TAT-peptides were dissolved in RPMI1640. Different concentrations of peptides were added to preseeded CNE2-EBV cells in a 12-well plate and incubated for 2 h at 37 °C. Cells were subsequently washed three times with PBS before being transferred to RPMI1640 supplemented with 10% FBS, penicillin/streptomycin, sodium butyrate, and PMA. After 48 h, the number of EBV genome copies in these cells were counted.

### EBV genome copy determination

CNE2-EBV cells pretransfected with TAT-peptide were treated with 2.5 mM sodium butyrate and 20 ng ml^−1^ phorbol 12-myristate 13-acetate (PMA) for 12 h to induce the production of EBV virions. After 48 h, the cells were collected and washed three times with PBS to measure viral replication, and the remaining medium was filtered through a 0.45-μm filter and centrifuged at 1000 × *g* for 10 min at 4 °C to remove the cell debries. The amount of encapsidated viral genome DNA was determined by a qPCR analysis of viral supernatants^61^. Briefly, the encapsidated viral genome DNA was extracted from induced cells by using a QIAamp DNA tissue kit (QIAGEN). The supernatant was digested with DNase I (105 U ml^−1^) at 37 °C for 1 h to degrade the naked EBV genome DNA before mixing it with lysis buffer and 0.1 mg ml^−1^ Proteinase K. Proteinase K was added to remove the viral envelope and capsid. The mixture was heated at 56 °C for 10 min, and then 75 °C for 20 min to deactivate the enzymes. The sample was diluted 1:10 by RNase-free water and subsequently applied to qPCR using primers for BALF5 DNA polymerase gene. Quantification of EBV-encoded genes was performed by qPCR using gene specific primers (Supplementary Table 5).

### Reporting summary

Further information on research design is available in the Nature Research Reporting Summary linked to this article.

## Supplementary information

Supplementary Information

Reporting Summary

## Data Availability

Data supporting the findings of this paper are available from the corresponding author upon reasonable request. The X-ray crystallographic coordinates and structure factor files have been deposited in the Protein Data Bank (PDB) under accession numbers 6LQN (BBRF2Δ) and 6LQO (BBRF2Δ-BSRF1Δ complex). Source data are provided with this paper.

## Source data

Source Data
