# Supplementary material for: Structure of Epstein-Barr virus tegument protein complex BBRF2-BSRF1 reveals its potential role in viral envelopment
Source: Nat Commun. 2020 Oct 26;11:5405. doi: 10.1038/s41467-020-19259-x (PMC7588443; doi:10.1038/s41467-020-19259-x)
Supplement: Supplementary file 1 — Supplementary Information [file 41467_2020_19259_MOESM1_ESM.pdf]

Supplementary Information for

Structure of Epstein-Barr virus tegument protein complex BBRF2-BSRF1 reveals its potential role in viral envelopment

Hui-Ping He[1,3], Meng Luo[1,3], Yu-Lu Cao[1], Yu-Xin Lin[1], Hua Zhang[1], Xiao Zhang[1], Jun-Ying Ou[1], Bing Yu[1], Xiaoxue Chen, Miao Xu[1], Lin Feng[1], Mu-Sheng Zeng[1], Yi-Xin Zeng[1], Song Gao[1,2,*]

Corresponding author: Song Gao (gaosong@sysucc.org.cn).

1. State Key Laboratory of Oncology in South China, Collaborative Innovation Center for Cancer Medicine, Sun Yat-sen University Cancer Center, Guangzhou, 510060, China.

2. Guangzhou Regenerative Medicine and Health Guangdong Laboratory, Guangzhou, 510530, China.

3. Equal contribution.

Supplementary Figures

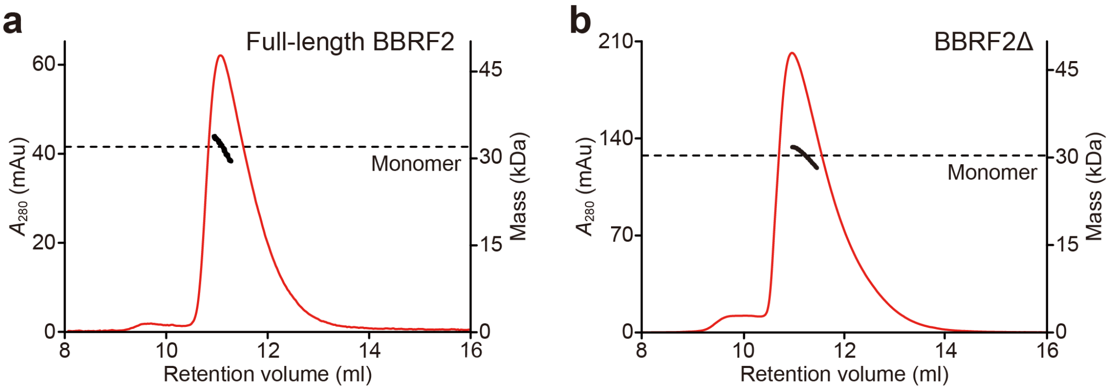

Supplementary Fig. 1: Full-length BBRF2 and BBRF2Δ are monomeric in solution

a and **b,** SEC-RALS analysis showing that full-length BBRF2 (**a**) and BBRF2Δ (**b**) are monomeric in solution, which are analyzed with a Superdex 75 SEC column. Calculated molecular masses at the absorption peak of 280 nm are plotted in black. mAU, milli-absorption units.

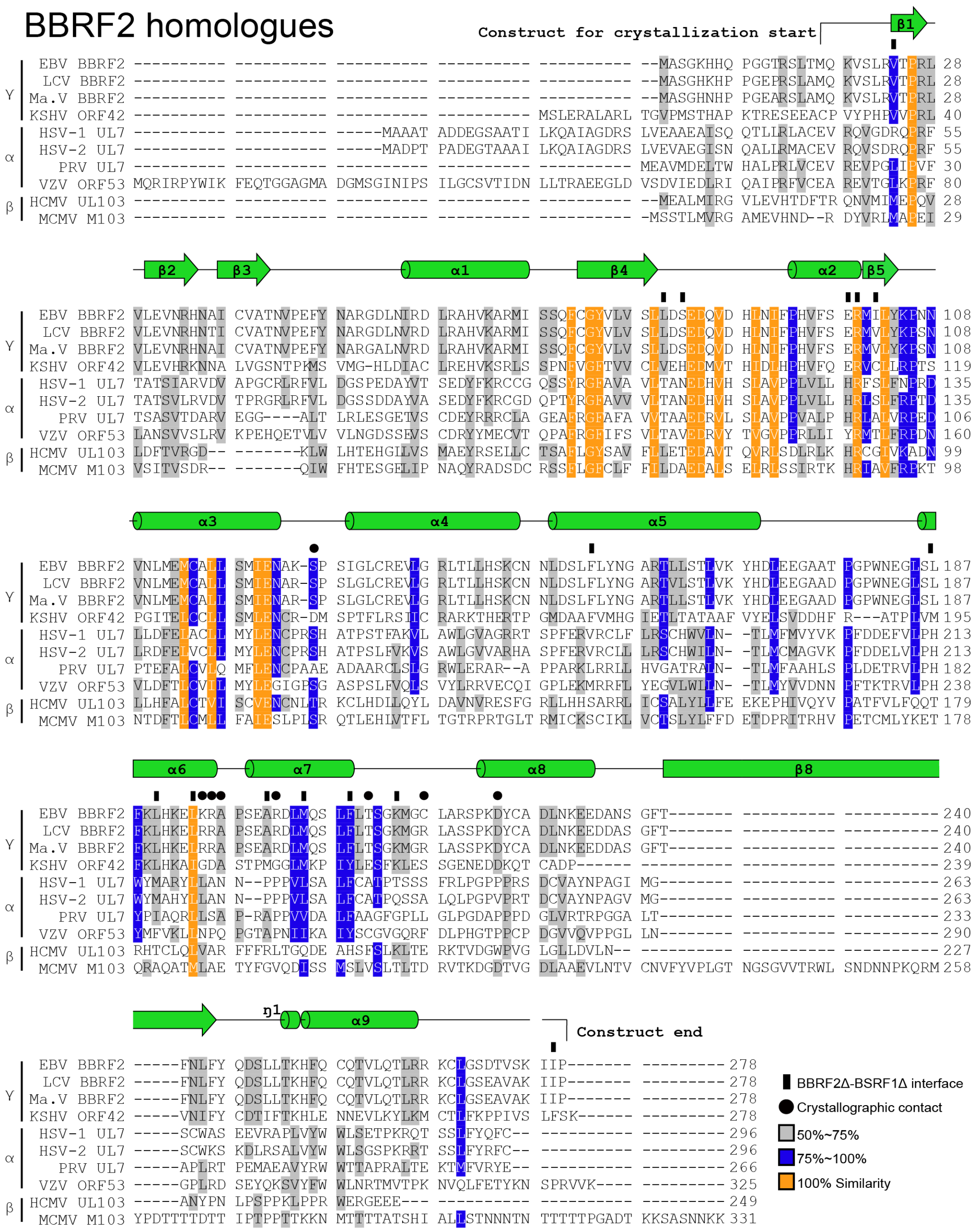

(Figure continued in next page)

Supplementary Fig. 2: Sequence alignments of BBRF2 and BSRF1 with their putative homologues in three herpesvirus subfamilies

Amino acid sequences of BBRF2 (UniProt accession P29882) and BSRF1 (P0CK49) with their putative homologues LCV (lymphocryptovirus *Macaca/pfe-lcl-E3*) BBRF2 (A0A0S0DWU3) and BSRF1 (A0A0S0DY77); Ma.V (*Macaca arctoides* gammaherpesvirus 1) BBRF2 (A0A3G1T4G0) and BSRF1 (A0A3G1T4E1); KSHV (Kaposi's sarcoma-associated herpesvirus) ORF42 (Q76RH1) and ORF55 (Q76rG2); HSV-1 (herpes simplex virus 1 UL7

(Legend continued in next page)

(Figure continuing)

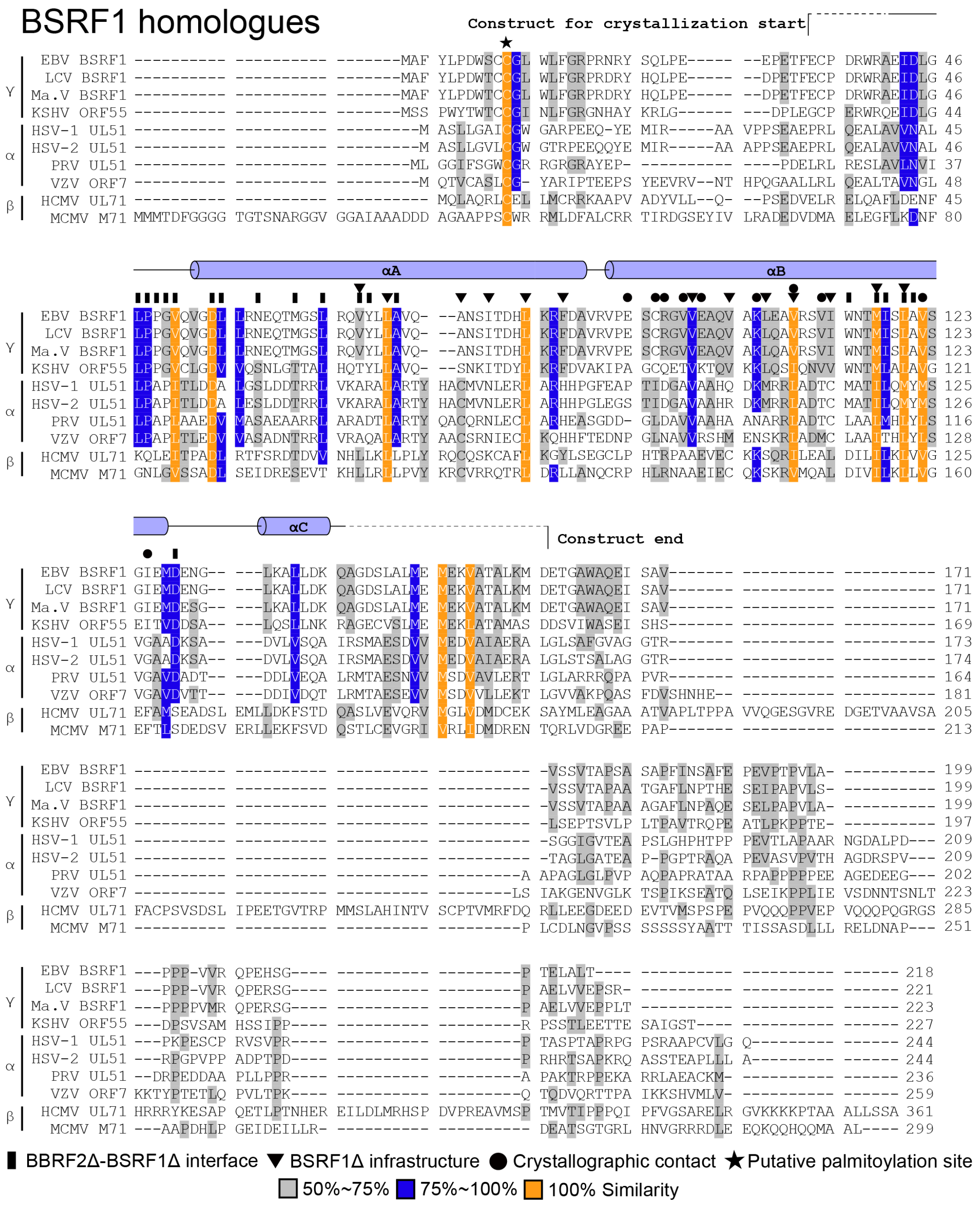

(Legend continuing)

(P10191) and UL51 (P10235); HSV-2 UL7 (W0NWT3) and UL51 (A0A0Y0R450), PRV, pseudorabies virus UL7 (G3G909) and UL51 (G3G8W4); VZV (varicella-zoster virus) ORF53 (Q6QCK3) and ORF7 (Q6QCP9); HCMV, (human cytomegalovirus) UL103 (P16734) and UL71 (P16823); MCMV (murine cytomegalovirus) M103 (Q69221) and M71 (Q69152). α-helices are shown as cylinders and β-strands as arrows. The secondary structure signs are coloured as in Fig. 4**b** and labelled as in Figs. 1**a** and 4**c**. Regions that are not have not been resolved in the crystal structure are indicated by dashed lines. Colour scheme of residue similarity is indicated. Residues are specified by different symbols according to their functions.

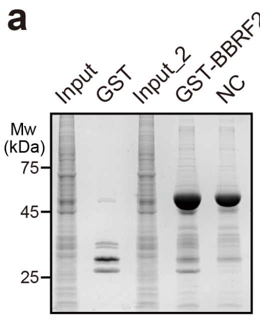

GST-BBRF2 purification

Protein	Peptide	Coverage (%)
BBRF2	12	59.7
BSRF1	1	6
MCP	2	2.3
BPLF1	3	1.6

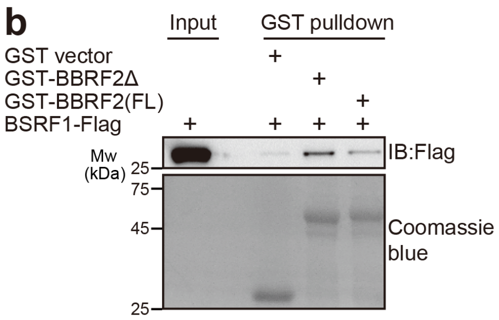

Supplementary Fig. 3: Interaction partners of BBRF2

a, Mass spectrometry analysis of BBRF2-associated proteins. GST pulldown was performed using HEK293T M81 cell extracts and GST-tagged BBRF2 purified from *E. coli*. GST-BBRF2: Purified GST-BBRF2 incubated with the cell lysates of HEK293T M81 cells; NC, negative control using purified GST-BBRF2 only (no cell lysates of HEK293T M81 cells). The major hits from the mass spectrometry analysis are listed. Source data are provided as a Source Data file.

b, Both full-length BBRF2 and BBRF2Δ interact with BSRF1. Source data are provided as a Source Data file. Each experiment was repeated three times independently with similar results.

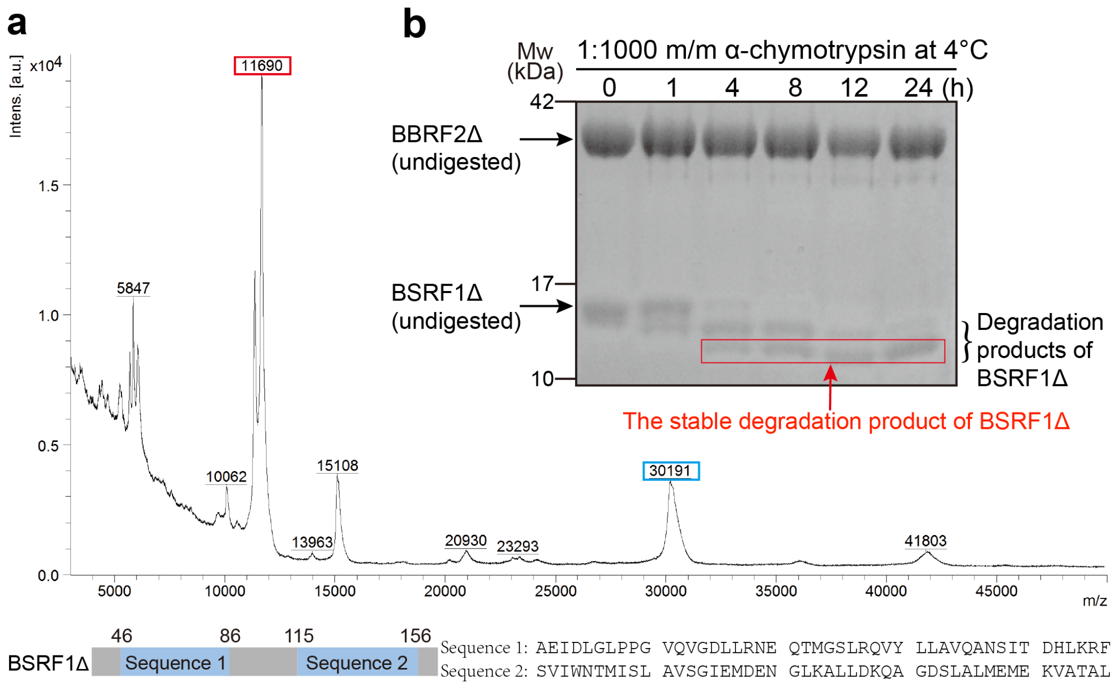

Supplementary Fig. 4: Limited proteolysis and mass spec analysis of BBRF2Δ-BSRF1Δ

a, The molecular masses of BBRF2Δ (blue) and BSRF1Δ (red) after α-chymotrypsin digestion measured by MALDI-TOF-MS. The post-digestion fragments of BSRF1Δ that have been identified by UHPLC-MS are shown below.

b, Time course of a limited proteolysis of the BBRF2Δ-BSRF1Δ mixture by α-chymotrypsin in 1:1000 m/m at 4°C. The bands of undigested and degraded BSRF1Δ, as well as undigested BBRF2Δ, are indicated. The stable degradation product of BSRF1Δ is specified. Source data are provided as a Source Data file.

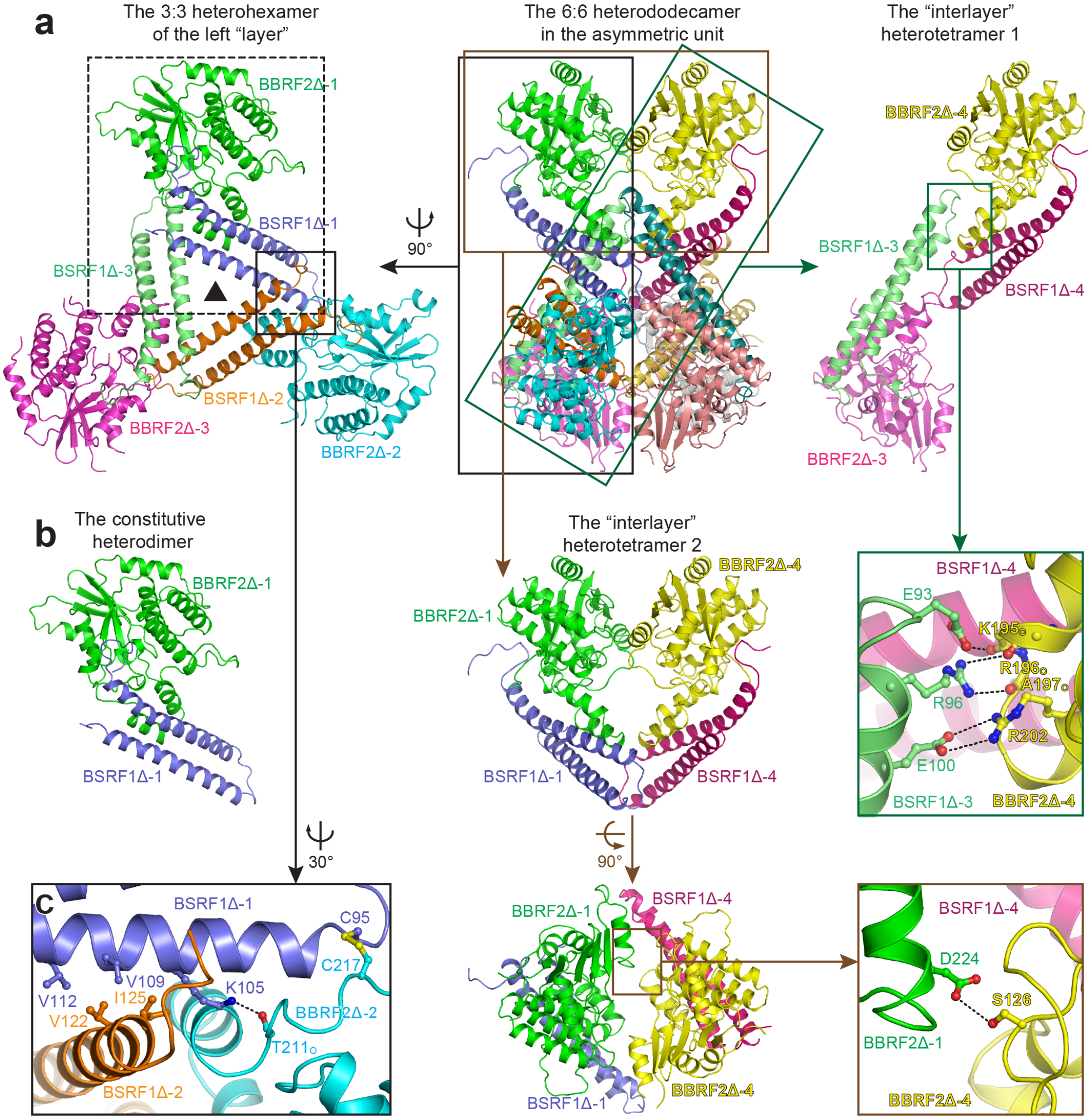

Supplementary Fig. 5: Assembly of the BBRF2Δ-BSRF1Δ complex in the crystal

a, Dissection of the 6:6 BBRF2Δ-BSRF1Δ heterododecamer in the asymmetric unit. Left: the 3:3 heterohexamer with 3-fold non-crystallographic symmetry. Right and lower: the interlayer association between the constitutive heterodimers. Molecules of BBRF2Δ and BSRF1Δ are differentially coloured and specified. Note the interfaces mediating the interlayer association of the constitutive heterodimers shown in the lower-right corner of the figure.

b, The constitutive BBRF2Δ-BSRF1Δ heterodimer.

c, The interface mediating the 3:3 BBRF2Δ-BSRF1Δ heterohexamer.

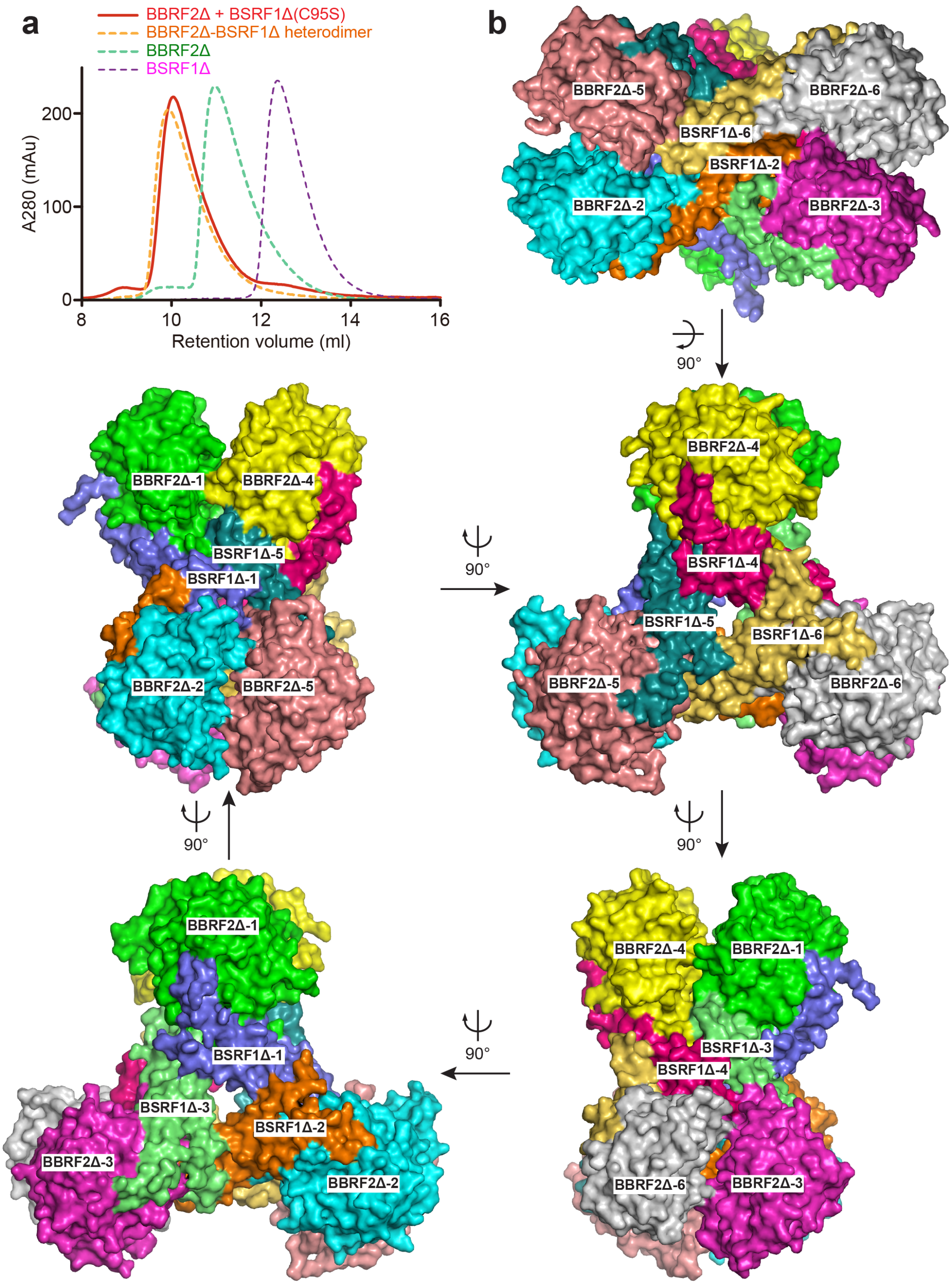

Supplementary Fig. 6: Assembly of the 6:6 BBRF2Δ-BSRF1Δ heterododecamer in the crystal

a, SEC analysis of co-incubated BBRF2Δ and BSRF1Δ(C95S) showing that BBRF2Δ efficiently forms heterodimer with BSRF1Δ(C95S) as with wild-type BSRF1Δ.

b, The positions of the component BBRF2Δ and BSRF1Δ molecules of the heterododecamer. Molecules of BBRF2Δ and BSRF1Δ are differentially coloured and specified as in Supplementary Fig. 5**a**.

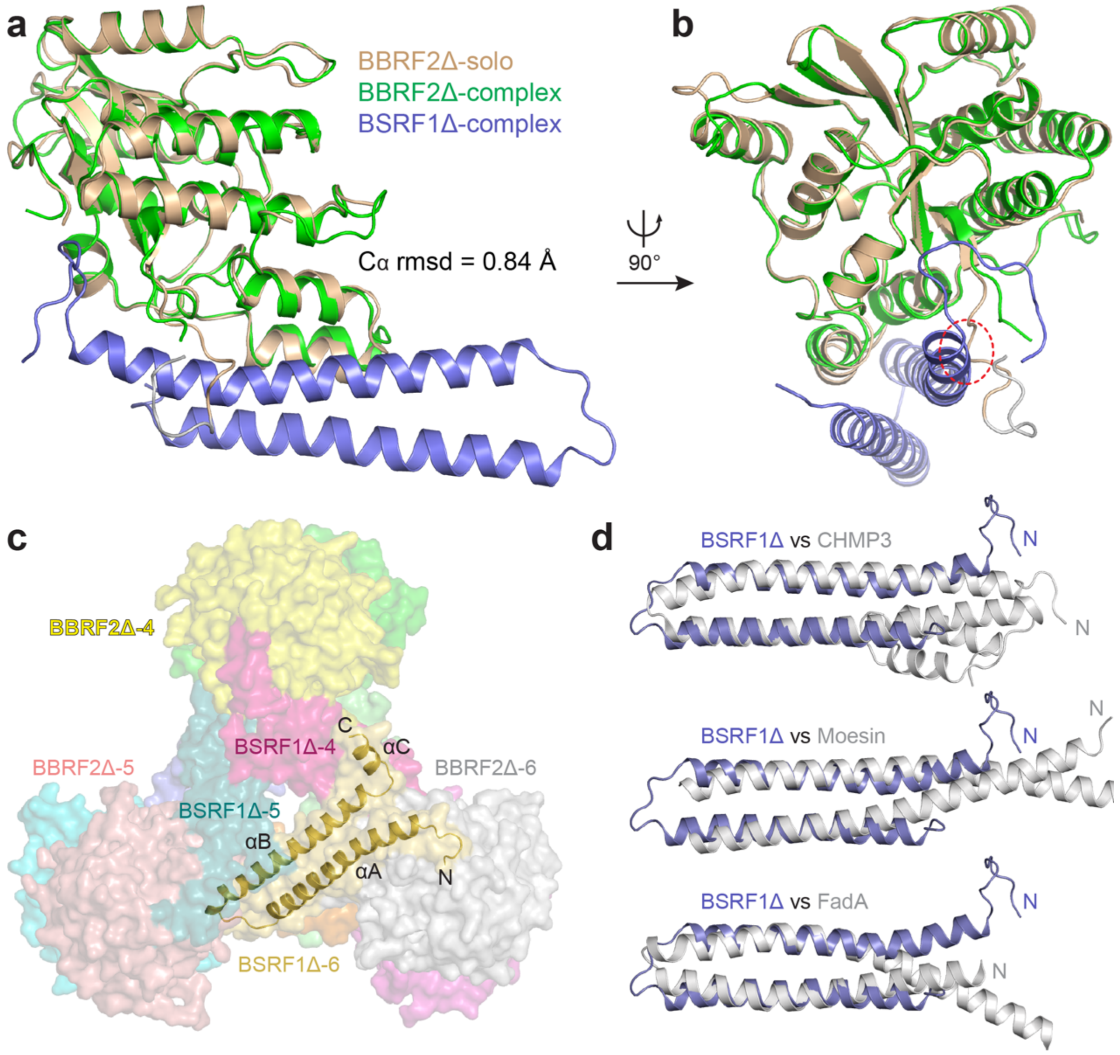

Supplementary Fig. 7: Structural feature of BSRF1Δ

a and **b,** Structural overlay of BBRF2Δ solo and BBRF2Δ-BSRF1Δ heterodimer in two views. The vector-encoding part of the N-terminal extension of BBRF2Δ solo is coloured grey. The clash between the N-terminus of BBRF2Δ in the solo structure and BSRF1Δ from the BBRF2Δ-BSRF1Δ heterodimer is indicated by a red dashed ellipse.

c, The BSRF1Δ molecule with the longest C-terminus as in the 3:3 BBRF2Δ-BSRF1Δ heterohexamer. The rest of the molecules are shown in transparency and coloured as in Fig. 3**a**. Note that the C-terminal αC of BSRF1Δ-6 does not interact with other molecules.

d, Structure overlay of BSRF1Δ to proteins with similar structures identified by the Dali server. CHMP3 (Protein Data Bank code 2GD5), Moesin (PDB code 2I1K), and FadA (3ETZ) are individually superimposed with BSRF1Δ.

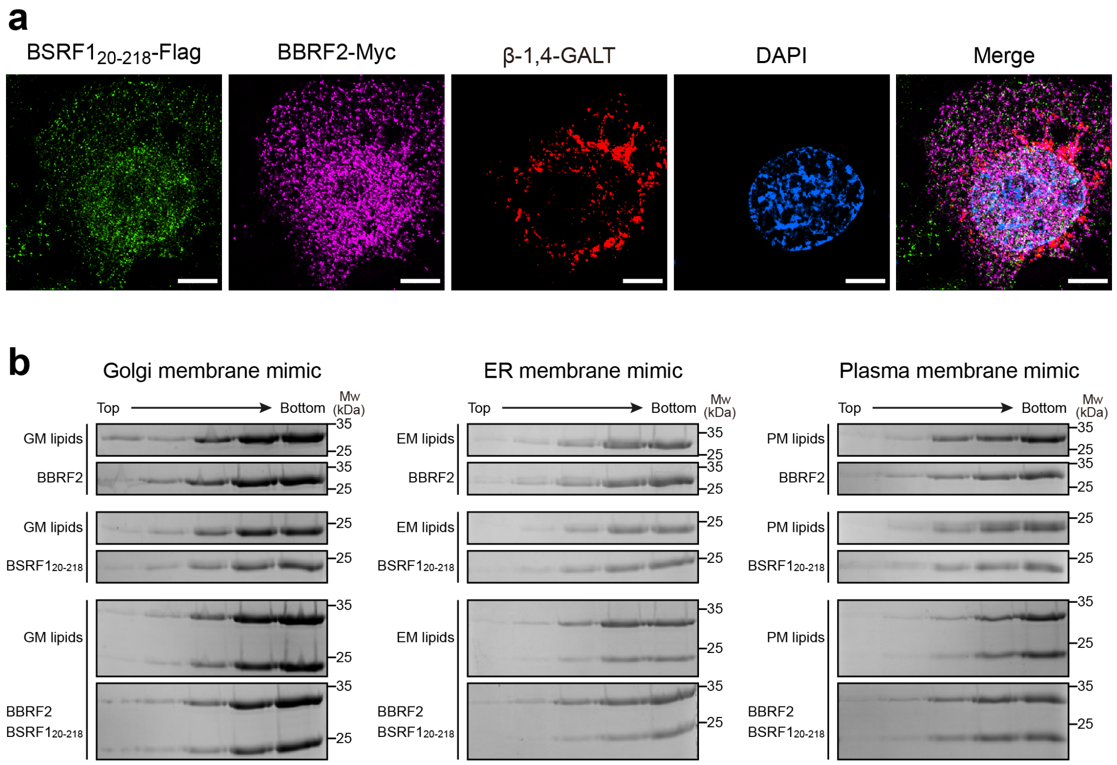

Supplementary Fig. 8: BSRF1 does not directly interact with lipids

a, Confocal immunofluorescence imaging of BSRF1$_{20\text{-}218}$ (green), BBRF2 full-length (purple), and β-1,4-GALT (red) in HeLa cells. Cell nuclei were counterstained with DAPI (blue). Scale bars, 5 μm.

b, Purified BBRF2, BSRF1$_{20\text{-}218}$, and BBRF2-BSRF1$_{20\text{-}218}$ complex were analyzed on gradients in the presence (top) or absence (bottom) of Golgi liposome mix, ER, and plasma liposome mix. Source data are provided as a Source Data file. Each experiment was repeated three times independently with similar results.

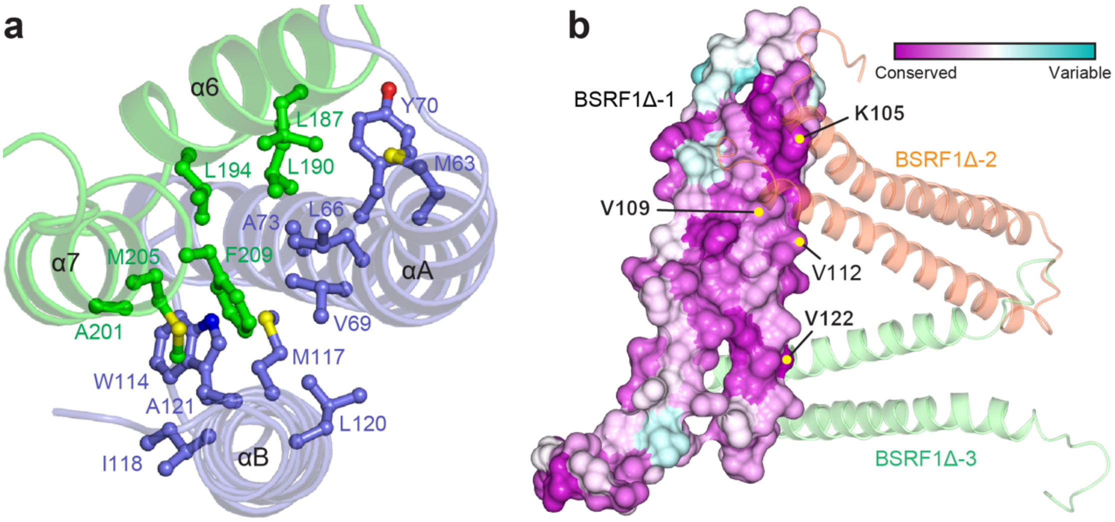

Supplementary Fig. 9: The BBRF2Δ-BSRF1Δ interfaces

a, The hydrophobic network that stabilizes the composite four-helix bundle of the BBRF2Δ-BSRF1Δ complex. Secondary structural elements are labelled. Involved residues are coloured as the molecule to which they belong.

b, Surface conservation plot of BSRF1Δ showing the conservation of the heterododecamer interface.

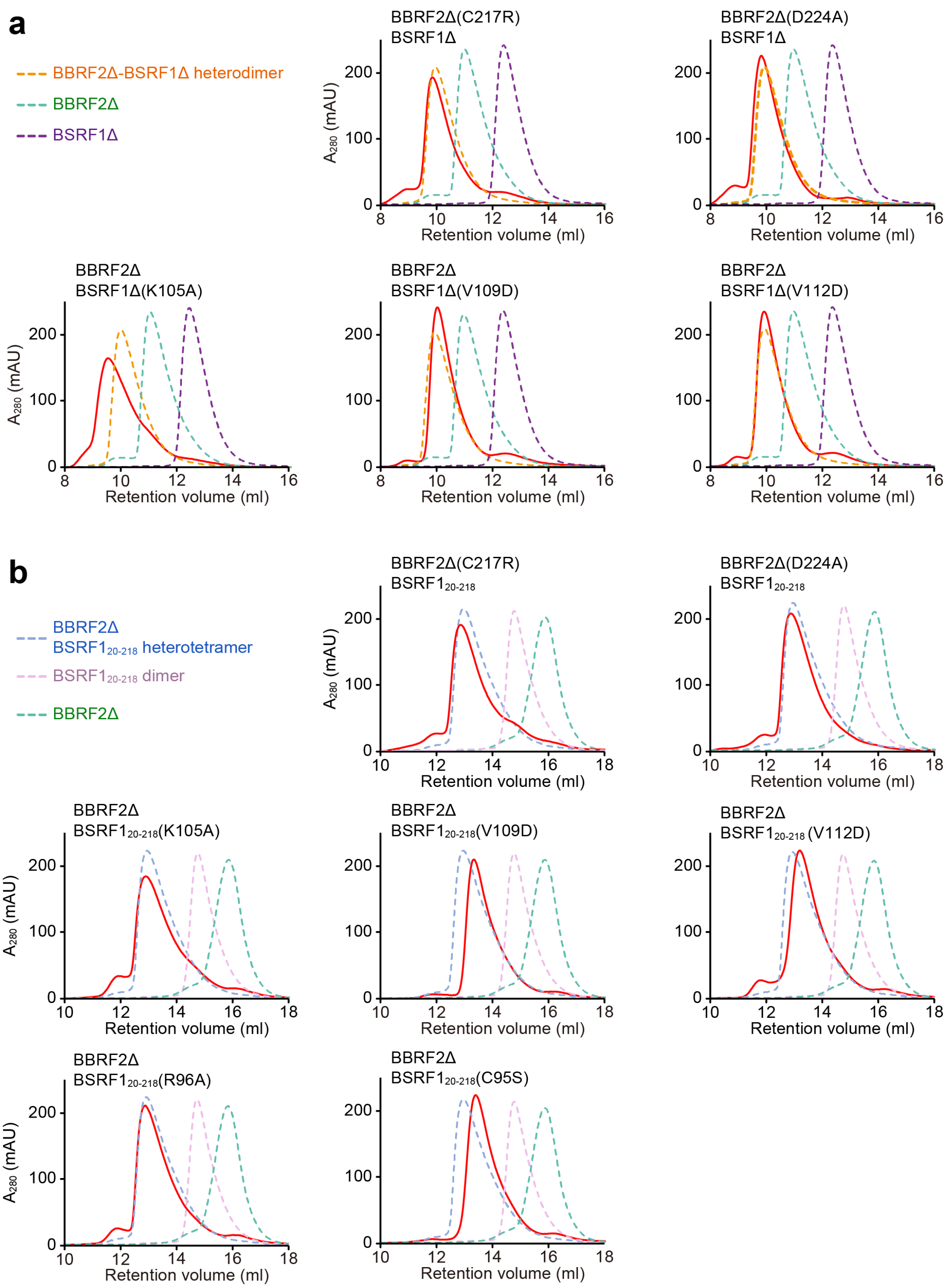

Supplementary Fig. 10: Characterization of the BBRF2-BSRF1 assembly interfaces

a, SEC analysis of the BBRF2Δ-BSRF1Δ interface mutants (red) using a Superdex 75 column. The elution peaks that are shown as dashed lines of the BBRF2Δ-BSRF1Δ heterodimer, BBRF2Δ monomer, and BSRF1Δ monomer are overlaid with each sample as references.

b, SEC analysis of the BBRF2Δ-BSRF1$_{20\text{-}218}$ interface mutants (red) using a Superdex 200 column. The elution peaks of the BBRF2Δ-BSRF1$_{20\text{-}218}$ heterotetramer (blue), BSRF1$_{20\text{-}218}$ dimer, and BBRF2Δ monomer are overlaid with each sample as references.

a

Peptide	Amino acid sequence	Position in BSRF1 (residue number)
P1	DLGLPPGVQVGDLLRNEQTMGSLRQVYLLAVQANSITD	44-81
P2	DLGLPPGVQVGD	44-55
P3	DLGLPPGVQVGDLLRNE	44-60
P4	EQTMGSLRQVYLLAVQANSITD	60-81
P5	RSVIWNTMISLAVSGIE	110-126

b

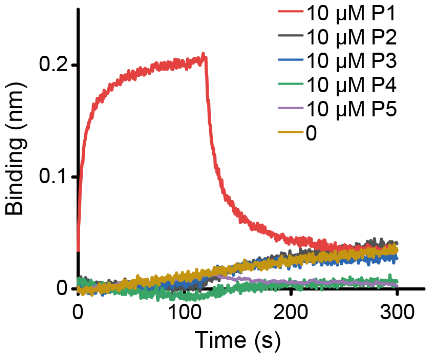

Supplementary Fig. 11: Screening BBRF2Δ-binding peptides

a, Sequences of five BSRF1-derived peptides.

b, BLI analysis of five peptides exposed to 10 μg ml^{-1} His$_6$-BBRF2Δ. Only P1 exhibits the binding for His$_6$-BBRF2Δ.

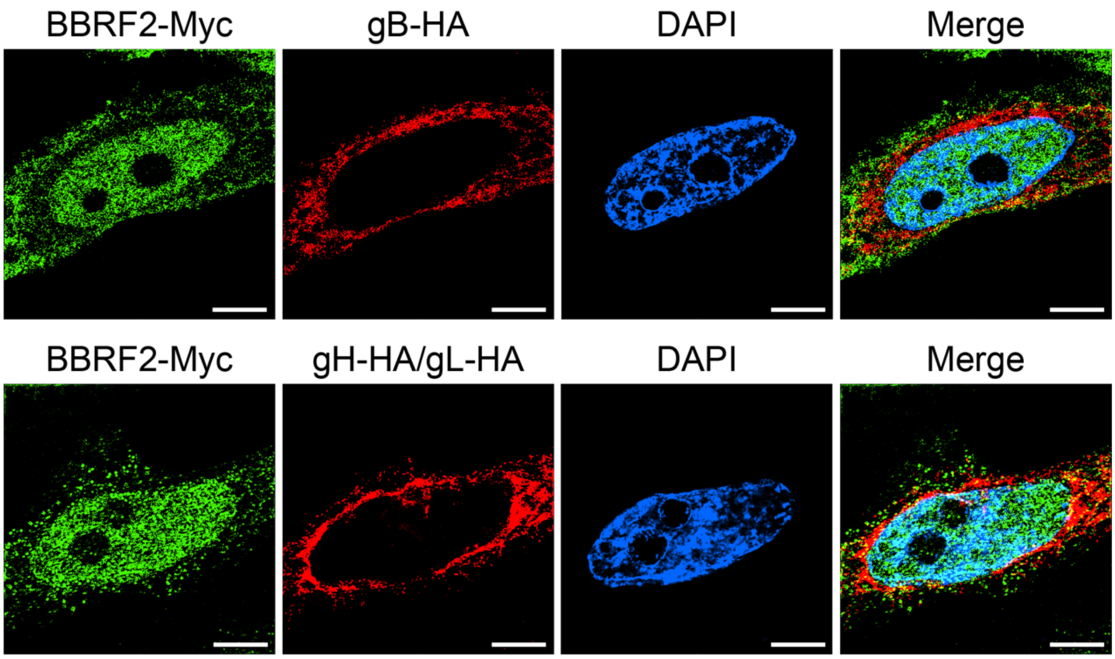

Supplementary Fig. 12: localization of co-expressed BBRF2 and EBV glycoproteins

Confocal immunofluorescence showing the localization of co-expressed BBRF2 (green) and EBV glycoproteins (red) in HeLa cells. Cell nuclei were counterstained with DAPI (blue). Scale bars, 5 μm. Each experiment was repeated three times independently with similar results.

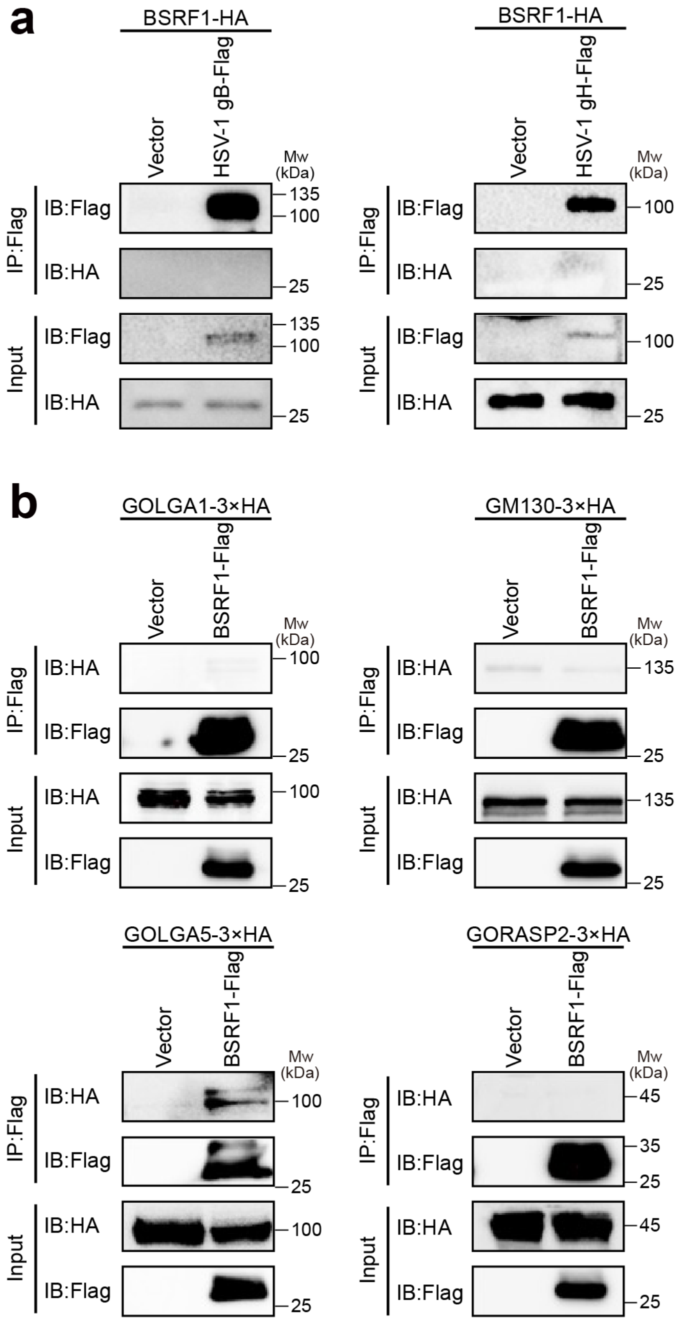

Supplementary Fig. 13: Co-IP of BSRF1 and HSV-1 glycoproteins/Golgi proteins

a, Co-IP of HA-tagged BSRF1 and Flag-tagged HSV-1 gB (left) or gH (right) in HEK293T cells. Source data are provided as a Source Data file.

b, Co-IP of four 3×HA-tagged Golgi proteins and Flag-tagged BSRF1 in HEK293T cells. Source data are provided as a Source Data file. Each experiment was repeated three times independently with similar results.

Supplementary Methods

Supplementary table 1: List of primers used for protein expression

Primer		Sequence 5'-3'
1	pET-28_6×His-BBRF2-FW	GGGCCCCATATGGGCGGATCCATGGCATCCGG CAAGCAC
2	pET-28_6×His-BBRF2-RE	GTGGTGGTGGTGGTGCTCGAGCTAGGGAATTA TTTTTGAGACCGTG
3	pET-28_6×His-BBRF2(17-278)-FW	GGGCCCCATATGGGCGGATCCATGCAAAAAGT AAGCCTGCGC
4	PGEX-6P-1_GST-BBRF2-FW	TCCAGGGGCCCCTGGGATCCATGGCATCCGGC AAGCAC
5	PGEX-6P-1_GST-BBRF2-RE	CACGATGCGGCCGCTCGAGCTAGGGAATTATTT TTGAGACCGTG
6	PGEX-6P-1_GST-BBRF2(17-278)-FW	TCCAGGGGCCCCTGGGATCCATGCAAAAAGTA AGCCTGCGC
7	pET-28_6×His-BSRF1(20-218)-FW	GGGCCCCATATGGGCGGATCCAGGAATAGATAC AGCCAGCTCCC
8	pET-28_6×His-BSRF1(20-218)-RE	GTGGTGGTGGTGGTGCTCGAGCTACGTTAACG CGAGCTCCG
9	PGEX-6P-1_GST- BSRF1(34-159)-FW	TCCAGGGGCCCCTGGGATCCGAGTGCCCGGA CCGCTGGC
10	PGEX-6P-1_GST- BSRF1(34-159)-RE	CACGATGCGGCCGCTCGAGCTAGTCCATCTTG AGCGCC

Supplementary table 2: List of primers used for site-directed mutagenesis

Primer for site-directed mutagenesis		
1	BBRF2(M205K)-FW	CGGGACCTCAAGCAGAGCCTCTTTCTGACC
2	BBRF2(M205K)-RE	GGTCAGAAAGAGGCTCTGCTTGAGGTCCCG
3	BBRF2(F209K)-FW	CAGAGCCTCAAACTGACCTCGGGGAAGATG
4	BBRF2(F209K)-RE	CATCTTCCCCGAGGTCAGTTTGAGGCTCTG
5	BBRF2(C217R)-FW	GATGGGGCGCCTGGCCAGGTCACCCAAGG
6	BBRF2(C217R)-RE	CCTTGGGTGACCTGGCCAGGCGCCCCATC
7	BBRF2(D224A)-FW	GTCACCCAAGGCTTACTGCGCGGATCTAAAC
8	BBRF2(D224A)-RE	GTTTAGATCCGCGCAGTAAGCCTTGGGTGAC
9	BSRF1(N59K)-FW	CTAAGAAAAGAGCAGACGATGGGCTCACTG
10	BSRF1(N59K)-RE	CAGTGAGCCCATCGTCTGCTCTTTTCTTAG
11	BSRF1(M63D)-FW	CTAAGAAATGAGCAGACGGATGGCTCACTG
12	BSRF1(M63D)-RE	CAGTGAGCCATCCGTCTGCTCATTTCTTAG
13	BSRF1(C95S)-FW	GTCCGCGTCCCTGAGAGCTCTCGTGGGGTG
14	BSRF1(C95S)-RE	CACCCCACGAGAGCTCTCAGGGACGCGGAC
15	BSRF1(R96A)-FW	GAGAGCTGTGCTGGGGTGGTGGAGGCCCAG
16	BSRF1(R96A)-RE	CTGGGCCTCCACCACCCCAGCACAGCTCTC
17	BSRF1(K105A)-FW	GTGGAGGCCCAGGTGGCCGCGCTTGAGGCC
18	BSRF1(K105A)-RE	GGCCTCAAGCGCGGCCACCTGGGCCTCCAC
19	BSRF1(V109D)-FW	GAGGCCGATCGCTCAGTCATCTGGAATACC
20	BSRF1(V109D)-RE	GGTATTCCAGATGACTGAGCGATCGGCCTC
21	BSRF1(V112D)-FW	CGCTCAGATATCTGGAATACCATGATCTC
22	BSRF1(V112D)-RE	GAGATCATGGTATTCCAGATATCTGAGCG

Supplementary table 3: List of primers used for co-IP/immunofluorescence

Primer		Sequence 5’-3’
1	pcDNA3.1_BBRF2-3×HA_FW	GGTGGAATTCTGCAGATATCATGGCATCCGGCAAGCACCATC
2	pcDNA3.1_BBRF2-3×HA_RE	GTATGGGTATCTAGACTCGAGGGGAATTATTTTTGAGACCG
3	pcDNA3.1_BBRF2(17-278)-3×HA_FW	GGTGGAATTCTGCAGATATCATGCAAAAAGTAAGCCTGCG
4	pDEST_Myc-BBRF2_FW	GGGGACAAGTTTGTACAAAAAAGCAGGCTCTATGGCATCCGGCAAGCACCA
5	pDEST_Myc-BBRF2_RE	GGGGACCACTTTGTACAAGAAAGCTGGGTACTAGGGAATTATTTTTGA
6	pcDNA3.1_BSRF1-Flag_FW	CTGGCTAGCAAGCTTGGATCCATGGCCTTCTATCTCCCAGAC
7	pcDNA3.1_BSRF1-Flag_RE	CATCCTTGTAGTCGAATTCCGTTAACGCGAGCTCCGTG
8	pcDNA3.1_BSRF1(20-218)-Flag_FW	GGCTAGCAAGCTTGGATCCAGGAATAGATACAGCCAGCTC
9	pcDNA3.1_BSRF1(20-218)-Flag_RE	CATCCTTGTAGTCGAATTCCGTTAACGCGAGCTCCGTGG
10	pcDNA3.1_MCP-Flag_FW	CTGGCTAGCAAGCTTGGATCCATGGCCTCAAATGAGGGTGTG
11	pcDNA3.1_MCP-Flag_RE	CATCCTTGTAGTCGAATTCAAAAACCACCTTATTTCCAAACTTTAATATTC
12	pcDNA3.1_BPLF1-1-Flag_FW	GGCTAGCAAGCTTGGATCCATGAGTAACGGCGACTGG
13	pcDNA3.1_BPLF1-1-Flag_RE	CATCCTTGTAGTCGAATTCGCTTGGGTCCTCAAGGTAGG
14	pcDNA3.1_BPLF1-2-Flag_FW	GGCTAGCAAGCTTGGATCCAGCCTCCCAACAGTGATGG
15	pcDNA3.1_BPLF1-2-Flag_RE	CATCCTTGTAGTCGAATTCTTTCGCCGGCCCTGCCG

16	pcDNA3.1_BPLF1-3-Flag_FW	GGCTAGCAAGCTTGGATCCTACGCCGGCACCC TCTGGC
17	pcDNA3.1_BPLF1-3-Flag_RE	CATCCTTGTAGTCGAATTCCAGATACAAAAACTT GAGTCTCTCGAG
18	pcDNA6B/gB-myc_FW	GTCCAGTGTGGTGGAATTCATGACTCGGCGTA GGGTGCTAAGC
19	pcDNA6B/gB-myc _RE	GGGCCCTCTAGACTCGAGAAACTCAGTCTCTG CCTCCCCAA
20	PCAGGS-HA-gB_FW	GGTACCCTCGAGATCGATATGACTCGGCGTAGG GTGCTAAGC
21	PCAGGS-HA-gB_RE	AGATCTGCTAGCACGCGTTTAAAACTCAGTCTC TGCCTCC
22	pcDNA6B/gH-myc _FW	GTCCAGTGTGGTGGAATTCATGCAGTTGCTCTG TGTTTTT
23	pcDNA6B/gH-myc _RE	GGGCCCTCTAGACTCGAGAAGGAAAAACATAA CAATCT
24	PCAGGS-HA-gH_FW	GGTACCCTCGAGATCGATATGCAGTTGCTCTGT GTTTTT
25	PCAGGS-HA-gH_RE	AGATCTGCTAGCACGCGTCTAAAGGAAAAACAT AACAA
26	pcDNA6B/gL-myc _FW	GTCCAGTGTGGTGGAATTCATGCGTGCTGTTG GTGTATTTC
27	pcDNA6B/gL-myc _RE	GGGCCCTCTAGACTCGAGGCCCCCGCGATGC CATGCGTATCTGT

Supplementary table 4: Sequence of BSRF1-derived peptides

Peptide	Sequence
P1	DLGLPPGVQVGDLLRNEQTMGSLRQVYLLAVQANSITD
P2	DLGLPPGVQVGD
P3	DLGLPPGVQVGDLLRNE
P4	EQTMGSLRQVYLLAVQANSITD
P5	RSVIWNTMISLAVSGIE
TAT-P1	YGRKKRRQRRRGGGSDLGLPPGVQVGDLLRNEQTMGSLR QVYLLAVQANSITD
TAT control	YGRKKRRQRRRAAQGFGCPL

Supplementary table 5: List of primers used for qPCR

Primer	Sequence
BBRF2	Forward: 5′-CTTATGGAGATGTGCGCCCT-3′
	Reverse: 5′-GTGGTACTTGACCAGGGTGG-3
BSRF1	Forward: 5′-TAAGCGGCATCGAGATGGAC-3′
	Reverse: 5′-AGTTGATGAAAGGGGCCGAG-3′
BALF5	Forward: 5′-GGTCACAATCTCCACGCTGA-3′
	Reverse: 5′-CAACGAGGCTGACCTGATCC-3′
